# Metabolic status and vascular endothelial structure in obese hypertensive patients treated with non-pharmacological therapies: A systematic review and meta-analysis

**DOI:** 10.1371/journal.pone.0279582

**Published:** 2022-12-30

**Authors:** Yingru Chen, Jie Yuan, Xueli Lei, Yan Cheng, Xijin Wei

**Affiliations:** 1 College of First Clinical Medicine, Shandong University of Traditional Chinese Medicine, Jinan, China; 2 Affiliated Hospital of Shandong University of Traditional Chinese Medicine, Jinan, China; Universidade de Sorocaba, BRAZIL

## Abstract

**Objective:**

This meta-analysis aimed to evaluate the efficacy of non-drug treatment on metabolism and vascular endothelium in obese hypertension.

**Methods:**

Relevant publications were searched in the PubMed, Embase, and Cochrane Library databases for clinical studies on the effects of non-pharmacological treatments in obese hypertensive patients published from inception to April 2022. After searching and screening the literature, information was extracted, and the quality of the literature was evaluated by the investigators. Data processing was performed using Rev Man 5.3 statistical analysis software, while the TSA 0.9 software was used for sequential analysis of blood pressure and endothelial-related indicators.

**Results:**

A total of 8 literature articles with 480 patients were included. The analysis showed that non-pharmacological treatment effectively reduced systolic blood pressure, diastolic blood pressure, heart rate, body weight, body mass index, glucose levels, soluble intercellular adhesion molecule 1, triglycerides, triglycerides, Low-density lipoprotein. For tumor necrosis factor α, soluble vascular cell adhesion molecule 1, high-density lipoprotein, C-reactive protein, high-sensitive C-reactive protein, and total antioxidant status by dietary supplements mainly. In contrast, no significant treatment effect was observed for Endothelin-1. Sequential analysis of the trial showed definitive evidence for improvement in blood pressure and inflammation.

**Conclusion:**

Non-pharmacological treatment of obese hypertensive patients may reduce blood pressure, body weight, and blood glucose, control inflammatory factor release and improve vascular endothelium to some extent.

## Introduction

Hypertension is one of the most important risk factors for cardiovascular disease, which seriously endangers human health [1]. About 13.9 million people worldwide suffer from hypertension, resulting in about 10.4 million premature deaths each year [2]. The prevalence of obesity has increased with the continuous changes in lifestyle, dietary habits, and working environment. In developed and developing countries, 100 million people are overweight or obese. By 2030, 38% of the world’s adults are expected to be overweight and another 20% obese [3, 4]. There is a close relationship between the causes of elevated hypertension in obese patients and the interaction between dietary, genetic, and environmental factors, but the pathophysiological mechanisms have not been clarified [5]. At present, thiazide diuretics, calcium channel blockers, angiotensin-converting enzyme inhibitors, and angiotensin receptor blockers are the main drugs for the treatment of hypertension. However, from 1990 to 2019, the global prevalence of hypertension has turned over, with a low drug control rate and treatment rate [6]. Furthermore, hypertension guidelines in various countries fail to provide targeted treatment strategy recommendations for hypertension in obese patients indicating the limitations of drug treatment. Therefore, non-pharmacological treatment modalities such as improving diet and increasing activity are highly valued in obese hypertensive patients [7]. The status of nutraceuticals and blood pressure control has been clearly presented in the position paper of the European Hypertension Association [8, 9].

The younger the age of onset or the more risk factors, the higher the incidence of cardiovascular disease and the risk of death in obese hypertensive patients. normal blood pressure, blood glucose, blood lipid, body weight, BMI, and other related indicators, are closely related to the risk of hypertension. In addition, vascular endothelial dysfunction is also an important pathological mechanism promoting the occurrence and development of hypertension and its related complications [10]. Therefore, early intervention for obese hypertensive patients should be based on lowering blood pressure, lowering blood lipids, controlling obesity, and improving endothelial function. Diverse exercise modalities and healthy dietary habits exert a positive effect on endothelial function improvement in obese hypertensive patients [11] and are also important for preventing target organ damage and improving the prognosis of hypertension. This study summarizes the RCTs on the non-pharmacological interventions, mainly including life-style changes and dietary supplements, for the treatment of obese patients with hypertension. This paper is the latest evidence-based evidence on hypertension in obese patients.

## Materials and methods

### The study protocol and registration

This protocol was written based on the preferred reporting items for systematic reviews and meta-analysis (PRISMA) guidelines [12]. The study was registered on the international prospective register of systematic reviews with registration number CRD42022326567 (URL: https://www.crd.york.ac.uk/prospero/#recordDetails).

### Inclusion criteria

(1) Study content: Randomized controlled trial (RCT) reporting on the effects of non-drug treatment on hypertensive vascular endothelium in obese patients and safety systematic review. (2) Study subjects: obese patients diagnosed with hypertension, including baseline demographics such as gender, age, race, etc. (3)Intervention measures: The control groups were given conventional treatment or other types of interventions. The test groups were given any treatment such as drugs under investigation, life-style changes, dietary habits, various psychological and behavioral habits, exercise for weight loss, or rehabilitation. (4)The outcome measures should includeat least one of the following. Primary outcome measures: systolic blood pressure(SBP), diastolic blood pressure(DBP), Heart rate, body weight, body mass index(BMI), glucose levels, forearm blood flow (FBF), soluble intercellular adhesion molecule 1(sICAM-1)levels, soluble vascular cell adhesion molecule 1(sVCAM-1) levels, endothelin-1(ET-1)levels, tumor necrosis factor α(TNF-α)levels, Secondary Outcome Measures: Low-density lipoprotein (LDL), high-density lipoprotein (HDL), triglycerides(TC), triglycerides(TG), C-reactive protein(CRP), high-sensitive C-reactive protein(hs-CRP), total antioxidant status (TAS).

### Exclusion criteria

Studies for which the full text could not be obtained and literature which did not use any of the above-mentioned evaluation indicators were excluded. Studies with incomplete data or containing serious errors were also excluded. For duplicate publications, only the first one was retained For studies sharing the same sets of data, only the one with the most complete data was retained.

### Data sources and search strategies

Relevant publications were searched in PubMed, Embase, and Cochrane Library databases. The search period spanned from database establishment to April 2022. Keywords included "Hypertension", "Weight Loss" and "randomized controlled trial", "Vascular endothelial". The search time was from database establishment to April 2022. The language of included kinds of literature was limited to English (S1 Table).

### Literature screening, extraction, and quality evaluation

Two investigators independently searched and screened the literature titles and abstracts, excluding obviously irrelevant studies. The remaining pieces of literature were screened by reading the full text. In case of any disagreement, a consensus was reached with the help of a third investigator. The extracted information mainly included: the first author, publication time, patient age, gender, number of included cases, specific intervention measures, and outcome measures. The Cochrane recommended risk of bias assessment tool was used to evaluate the quality of the included pieces of literature.

### Statistical processing

The Meta-analysis of the collected data was performed using Review Manager 5.3 software and sequential analysis was performed with TAS V0.9 software. The heterogeneity of the included trials was analyzed by the Q test, and the heterogeneity was judged by the *I*^*2*^ test. *I*^*2*^ < 50%, indicated no significant heterogeneity in the included literature, and the fixed-effect model was selected for analysis. In contrast, *I*^*2*^ ≥ 50%, indicated greater heterogeneity and the random-effect model was applied. *p* < 0.05was considered, statistically significant. Moreover, a funnel plot was used to evaluate publication bias. The results were then subjected to TAS.

## Results

### Literature search results

**Fig 1:**
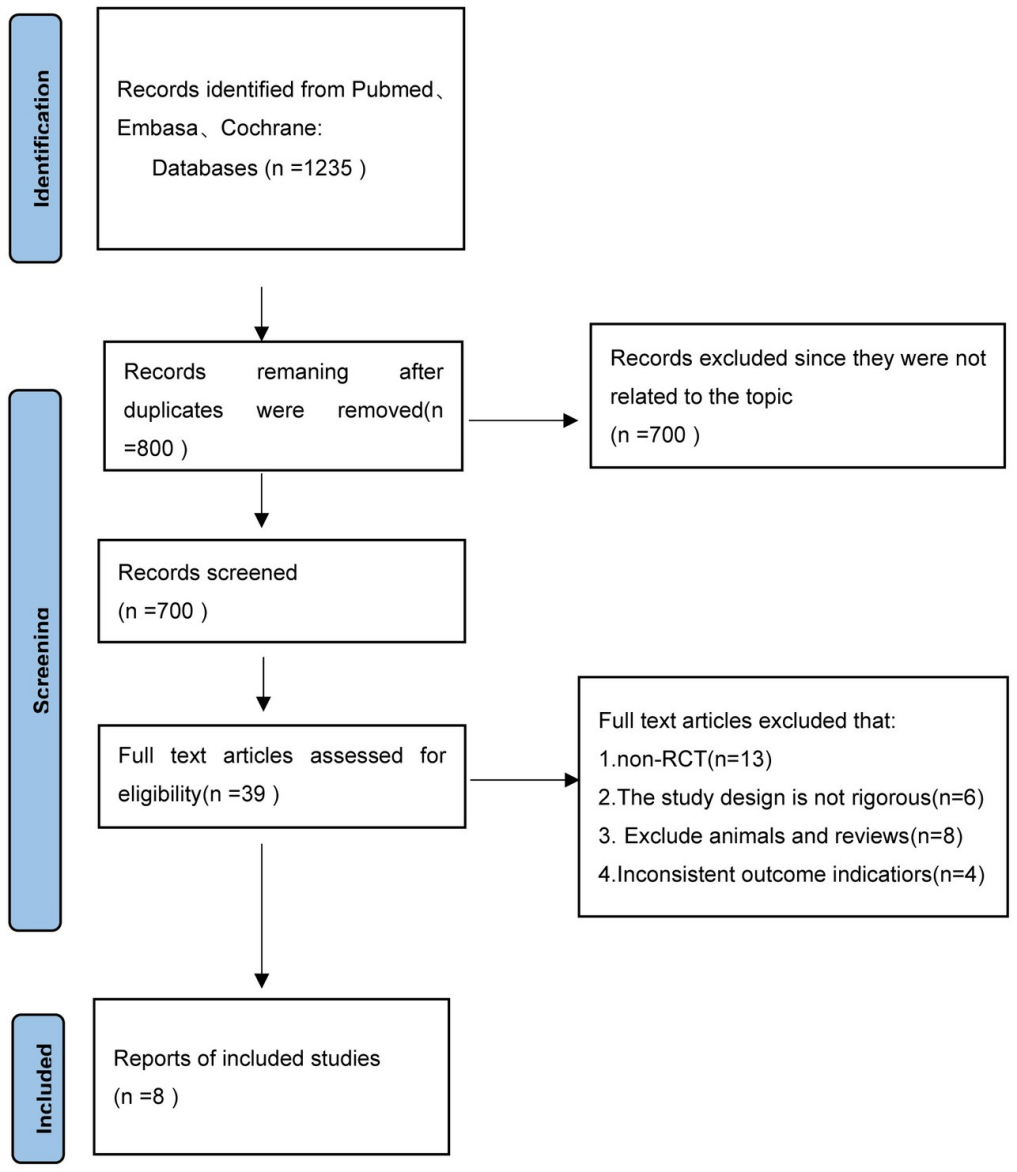
Literature screening flow chart.

A total of 1235 relevant articles were obtained after the initial search, 800 articles remained after excluding removing duplicate publications, and 39 articles were obtained after further reading the abstracts and full texts. In total, 13 articles that were not RCTs, 6 articles that were not rigorously designed, 8 animal trials, and 4 articles within consistent outcome measures were excluded. Finally, 8 pieces of literature [13–20] were included in the meta-analysis. Fig 1, illustrates the literature retrieval and flow chart.

### Risk of bias

**Fig 2:**
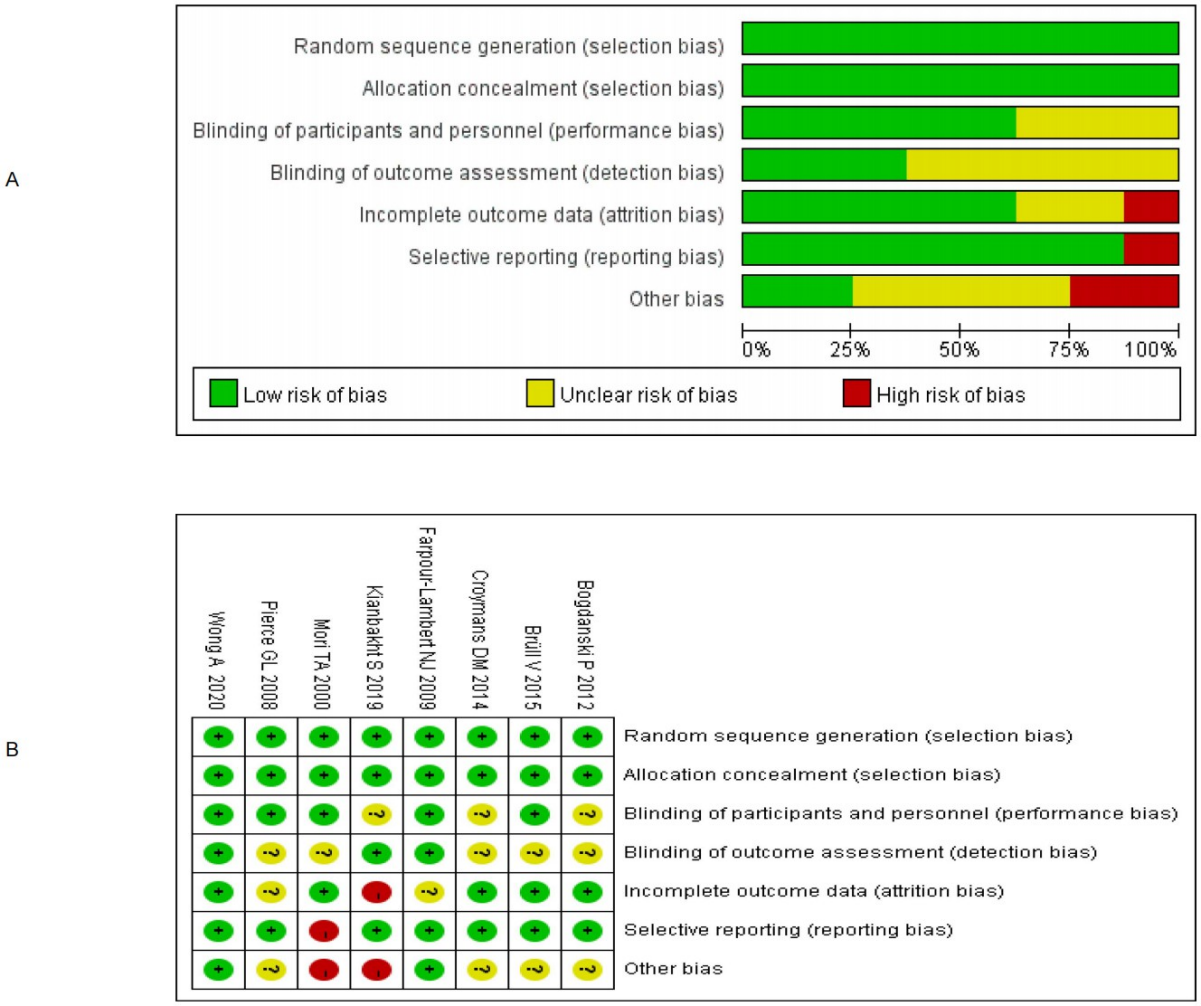
Bias risk charts for the included studies.

According to the risk of bias assessment tool recommended by Cochrane, there were 3 articles with low bias and 5 articles with moderate bias, as shown in Fig 2.

### Basic information of included literature

Eight pieces of literature with a total of 480 subjects were included in this study, of which 262 subjects received non-drug treatment, and 218 subjects had no intervention. The patients were from different countries around the world. The specific main study characteristics are summarized in Table 1 below.

**Table 1:** Basic information of the included literature.

Author, Year	Cases		Age (years)		Treatment		Intervention period /month	Outcome indicator
	T	C	T	C	T	C		
Mori TA, 2000 [15]	26	14	50.8±2.0	50.6±1.4	EPA/DHA	Placebo	1.5	SBP, DBP, Bodyweight, BMI, TNF-α, LDL, HDL, TC, TG, Glucose, CRP, TAS
Pierce GL, 2008 [16]	26	14	49.2±2.5	40.8±3.3	Weight Loss	Untreated	4	SBP, DBP, Heart rate, Body weight, BMI, ET-1, TNF-α, LDL, HDL, TC, TG, Glucose
Farpour-Lambert NJ, 2009 [17]	22	22	9.1±1.4	8.8±1.6	Obese Exercise	Untreated	3	SBP, DBP, Body weight, BMI, LDL, HDL, TC, TG, FBF
Bogdanski P, 2012 [13]	28	28	49.2±8.8	51.5±7.4	Green tea extract	Placebo	3	SBP, DBP, Body weight, BMI, LDL, HDL, TC, TG, Glucose, FBF
Croymans DM, 2014 [18]	28	8	22.0	21.5	resistance training	Untreated	3	SBP, DBP, Heart rate, Body weight, BMI, sICAM-1, sVCAM-1, LDL, HDL, TG, CRP
Brüll V, 2015 [14]	68	68	7.4±10.5	59.6±4.3	Quercetin	Placebo	1.5	SBP, DBP, Heart rate, Body weight, BMI, sICAM-1, sVCAM-1, LDL, HDL, TG, CRP
Kianbakht S, 2019 [20]	50	50	57±6	61±8	V. Arctostaphylos berry extract	Placebo	3	SBP, DBP, Heart rate, sICAM-1, sVCAM-1, ET-1, LDL, HDL, TG, CRP
Wong A, 2020 [19]	14	14	22±1	23±1	Placebo	Untreated	3	SBP, DBP, BMI

Note: T: The treatment group; C: Control group;

### Meta-analysis results

#### Primary outcome measures

**Fig 3:**
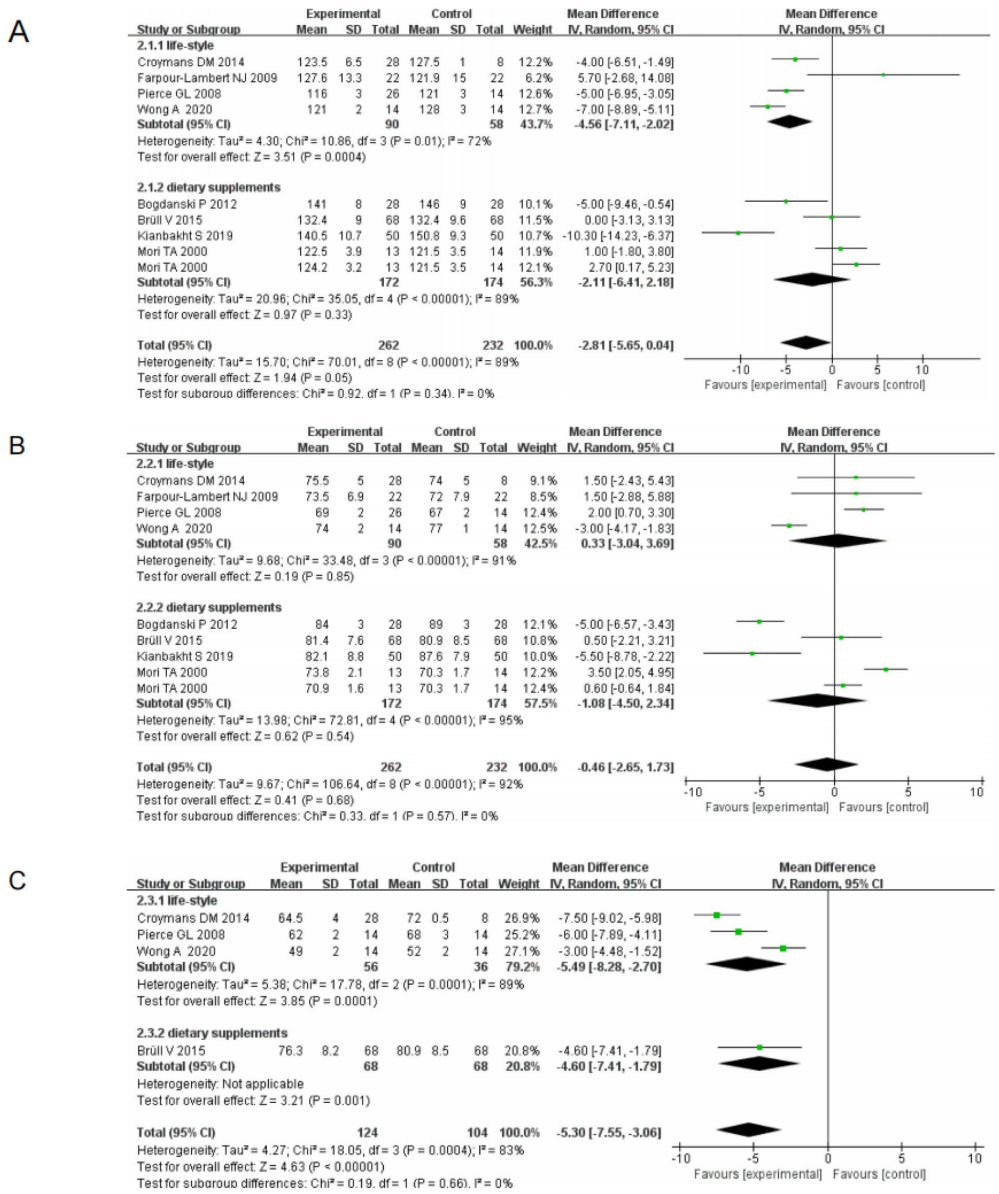
Forest plot of primary outcome indicators(I) (A) SBP Forest plot of primary outcome indicators (B) DBP Forest plot of primary outcome indicators (C) Heart rate Forest plot of primary outcome indicators.

**Fig 4:**
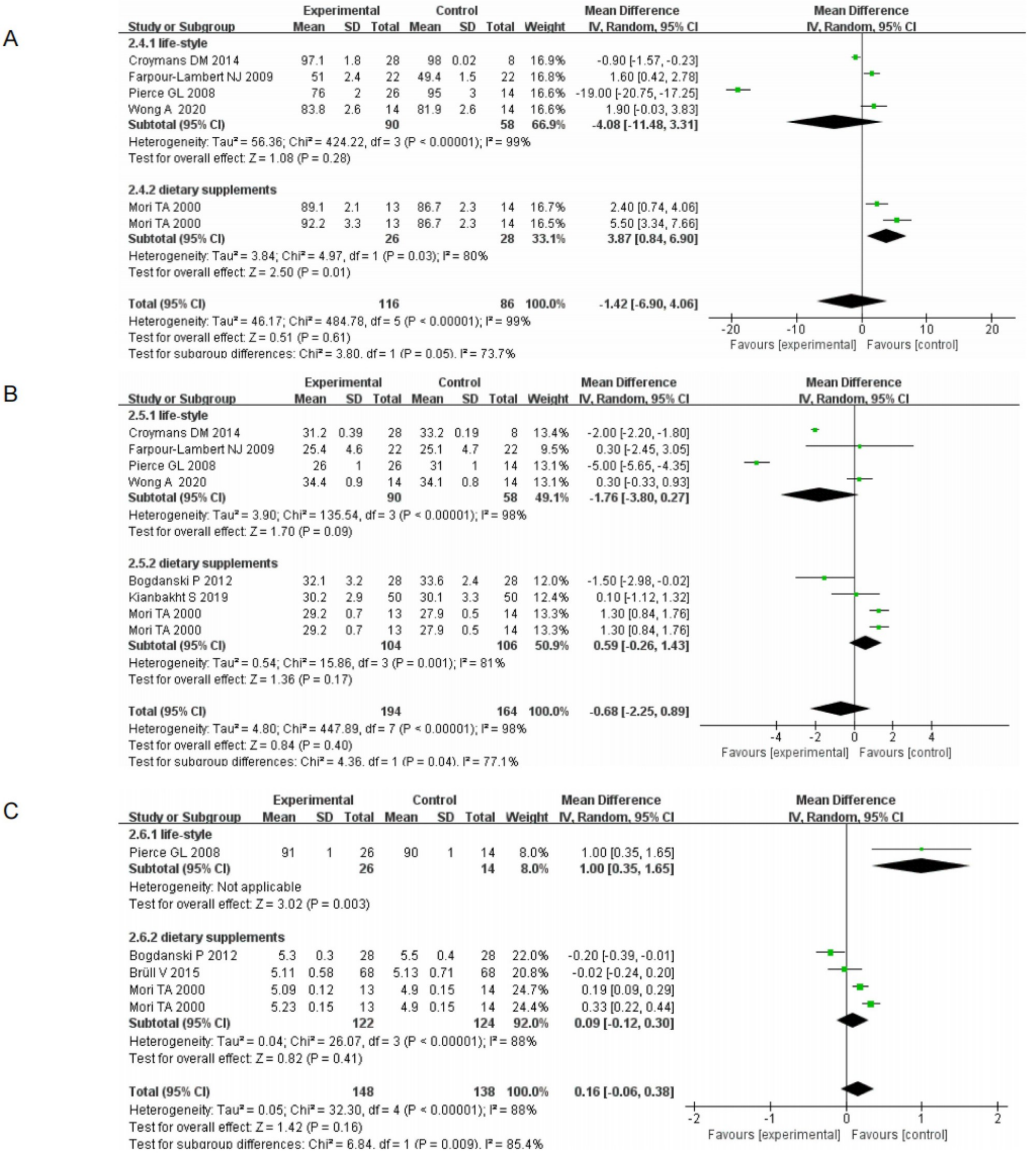
Forest plot of primary outcome indicators(II) (A) Boby weight Forest plot of primary outcome indicators (B) BMI Forest plot of primary outcome indicators (C) Glucose Forest plot of primary outcome indicators.

(I) Subgroup analysis based on life-style and dietary supplements showed that improvements in SBP were observed in 43.7% of cases with lifestyle changes (95% CI[-7.11, -2.02], I^2^ = 72%, *p* = 0.01) and 56.3% of cases with dietary supplements (95% CI[-6.41, 2.18], I^2^ = 89%, *p*<0.00001). DBP improvements were found in 42.5% of patients with life-style changes (95% CI[-3.04, 3.69], I^2^ = 91%, *p* < 0.00001) and 57.5% of patients with dietary supplements (95% CI[-4.50, 2.34], I^2^ = 95%, *p* <0.00001). Improvements in heart rate were observed in 89% of cases with life-style changes (95% CI[—8.28, -2.70], I^2^ = 89%, *p* = 0.00001), and in 20.8% of patients with dietary supplements (95% CI[-7.41, -1.79], *p* = 0.001). All analyses were performed using a random effects model, with the statistical significance set at *p* <0.05 (Fig 3). Therefore, this analysis demonstrated that non-pharmacological treatment was effective in reducing systolic pressure, diastolic pressure and heart rate.

(II) Body weight by life-style and dietary supplements: 66.9%, (95% CI[-11.48, 3.31], I^2^ = 99%, *p* <0.00001); 33.1%, (95% CI[0.84, 6.90], I^2^ = 80%, P<0.00001) respectively. BMI by life-style and dietary supplements were: 49.1%, (95% CI[-3.80, 0.27], I^2^ = 98%, *p* <0.00001); 50.9%, (95% CI[-0.26, 1.43], I^2^ = 81%, *p* = 0.001); Glucose by life-style and dietary supplements were: 8%, (95% CI [0.35, 1.65], *p* = 0.003); 92%, (95% CI [-0.12, 0.30], I^2^ = 81%, *p* <0.00001) respectively. All analyses were performed using a random effects model, with the statistical significance set at *p* <0.05 (Fig 4). The results demonstrated that non-pharmacological treatment was effective in improving weight, BMI and glucose indicators in subjects.

(III): One article [15] measured forearm blood flow in subjects, revealing an association between non-drug treatment and forearm vasodilation due to endogenous and exogenous NO donors, which attenuated the vasoconstrictor response to norepinephrine. Three pieces of literature [14, 15, 19] demonstrated improvements in endothelium-dependent vasodilation (EDD) of peripheral ductal arteries and resistance vessels, as well as brachial artery flow-mediated dilatation(FMD) and forearm blood flow to acetylcholine, should be improved on both sides. The improvement in vascular endothelial function may be attributed to the reduced abdominal visceral fat and the decrease in lipoprotein after weight loss, which could decrease oxidative stress and increase NO bioavailability to improve EDD.

**Fig 5:**
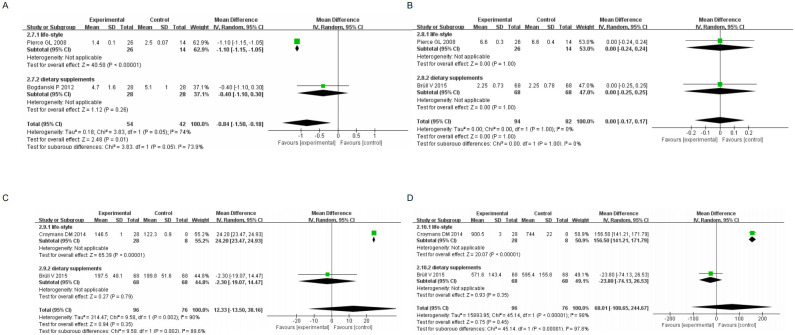
Forest plot of primary outcome indicators(III) (A) TNF-α Forest plot of primary outcome indicators (B) Endothelin-1 Forest plot of primary outcome indicators (C) sICAM-1 Forest plot of primary outcome indicators (D) sVCAM-1 Forest plot of primary outcome indicators.

TNF-α by life-style and dietary supplements were: 62.9%, (95% CI[-1.15, -1.05], *p* <0.00001); 37.1%, (95% CI[-1.10, 0.30], *p* = 0.26); Endothelin-1 by life-style and dietary supplements respectively: 53%, (95% CI[-0.24, 0.24], *p* = 1.00); 47%, (95% CI[-0.25, 0.25], *p* = 1.00); sICAM-1 as life-style and dietary supplements respectively: 55.2%, (95% CI [23.47, 24.93], *p* <0.00001); 44.8%, (95% CI [-19.07, 14.47], *p* = 0.79); and sVCAM-1 as life-style and dietary supplements were: 50.9%, (95% CI [141.21, 171.79], *p* <0.00001); 49.1%, (95% CI [-74.13., 26.53], *p* = 0.35). As shown in Fig 5, all the analyses were carried out using a random effects model. TNF-α, sVCAM-1 predominantly dietary supplements and Endothelin-1 by life-style and dietary supplements analyses showed *p* = 0.26, *p* = 0.35, *p* = 1.00, *p* = 1.00, *p* >0.05, indicating no significant effect of treatment. The remaining results showed *p* <0.05, indicating a significant effect of non-pharmacological treatment.

#### Secondary primary outcome measures

**Fig 6:**
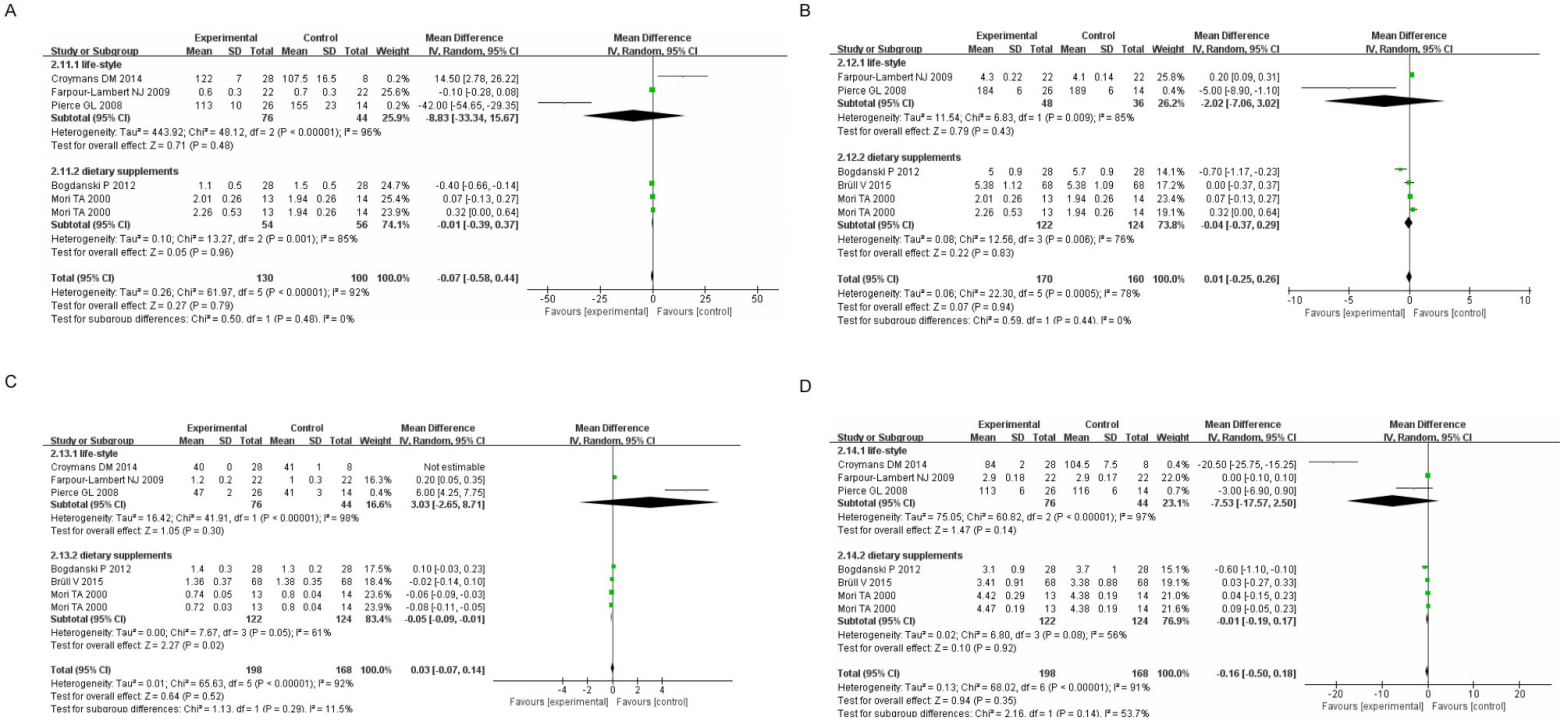
Forest map secondary outcome indicators (A) TG Forest map secondary outcome indicators (B) TC Forest map secondary outcome indicators (C) HDL Forest map secondary outcome indicators (D) LDL Forest map secondary outcome indicators.

**Fig 7:**
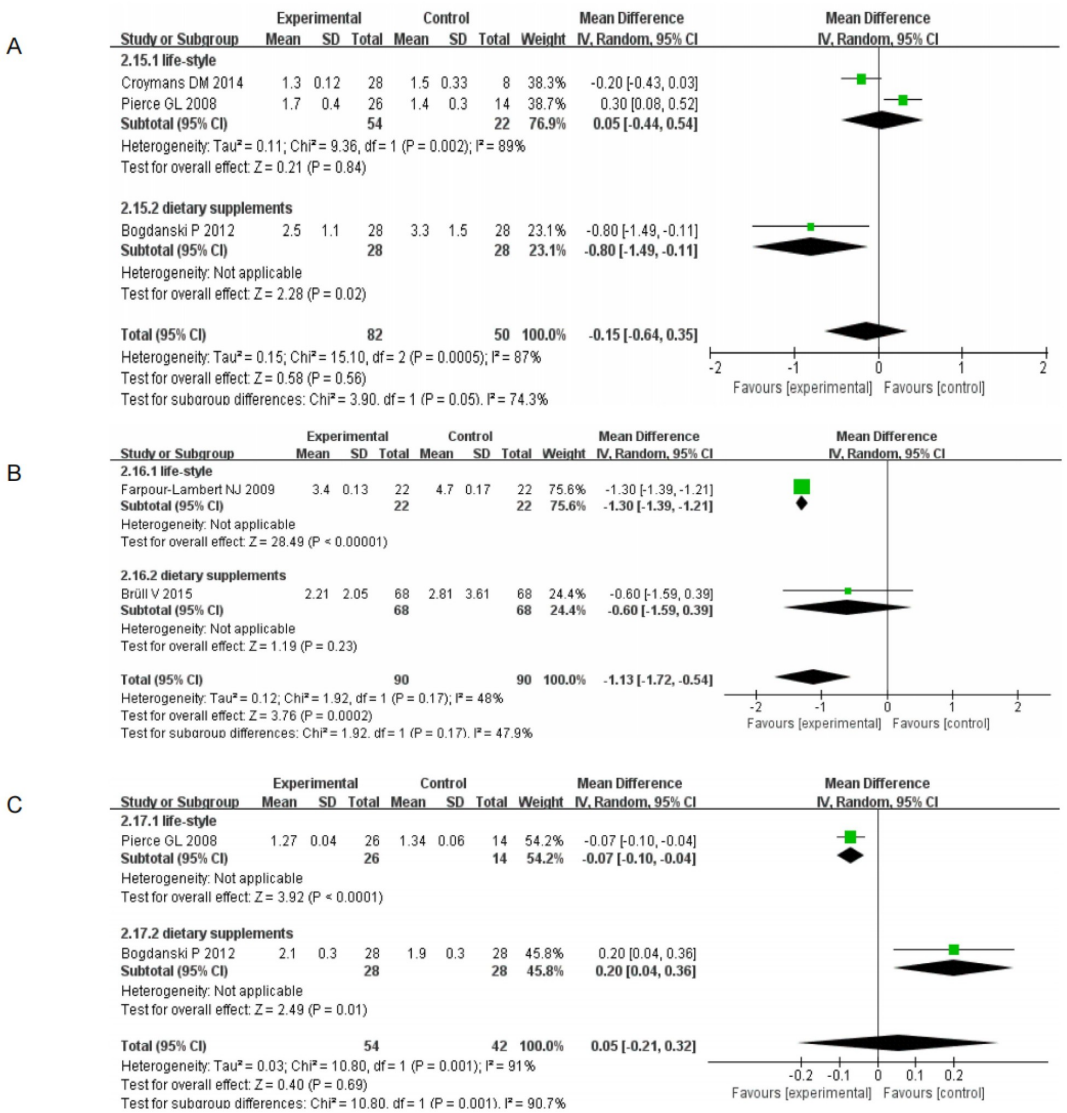
Forest map secondary outcome indicators (A) CRP Forest map secondary outcome indicators (B) hs-CRP Forest map secondary outcome indicators (C) TAS Forest map secondary outcome indicators.

TG as lifestyle and dietary supplements were: 25.9%, (95% CI[-33.34, 15.67], I2 = 96% *p* <0.00001); 74.1%, (95% CI[-0.39, 0.37], I^2^ = 92%, *p* = 0.001); TC as lifestyle and dietary supplements were: 26.2%, (95% CI [-7.06, 3.02], I^2^ = 85% P = 0.009); 73.8%, (95% CI [-0.37, 0.29], I^2^ = 76% *p* = 0.006); HDL as lifestyle and dietary supplements were: 16.6%, (95% CI [-2.65, 8.71], I^2^ = 98% *p* <0.00001); 83.4%, (95% CI [-0.09, -0.01], I2 = 61%, *p* = 0.05); LDL as lifestyle and dietary supplements 23.1%, (95% CI[-17.57, 2.5], I^2^ = 97% *p* <0.00001); 76.9%, (95% CI[-0.19, 0.17], I^2^ = 56%, *p* = 0.008); CRP by lifestyle and dietary supplements respectively: 76.9%, (95% CI[-0.44, 0.54], I^2^ = 89%, *p* = 0.002); 23.1%, (95% CI[-1.49, -0.11], *p* = 0.02); hs-CRP as lifestyle and dietary supplements were: 75.6%, (95% CI[-1.39, -1.21], *p* <0.00001); 24.4%, (95% CI[-1.59, 0.39], *p* = 0.23); TAS by lifestyle and dietary supplements: 54.2%, (95% CI[-0.10, -0.04], *p* <0.00001); 45.8%, (95% CI[-0.04], *p* <0.00001); and 45.8%, (95% CI [-0.04, 0.36], *p* = 0.01). As displayed in Figs 6 and 7, all analyses were performed with a random effects model, where HDL, CRP, hs-CRP and TAS showed *p* = 0.05, *p* = 0.02, *p* = 0.23 and *p* = 0.01 with dietary supplements, respectively. The differences were not statistically significant, indicating no significant treatment effect. The other indicators showed a statistically significant difference at *p* <0.05, indicating a possible non-pharmacological treatment effect.

#### Risk of bias assessment

**Fig 8:**
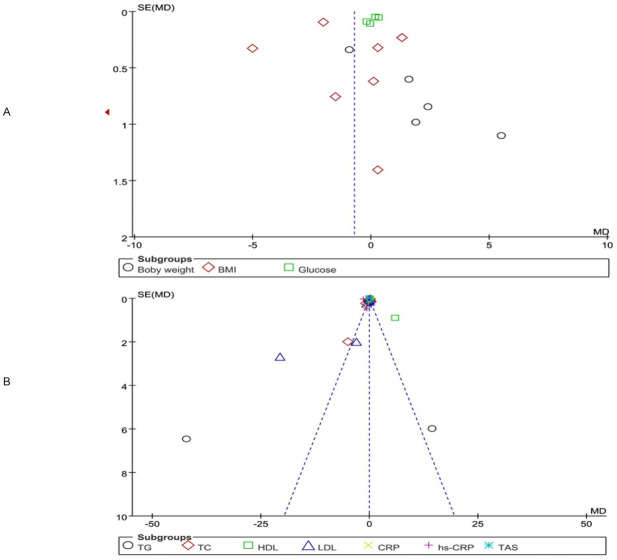
Risk of bias assessment (A) Funnel plot of body weight, BMI, and blood glucose (B) Funnel plot of inflammatory factors.

Publication bias was assessed for the primary outcome measures of weight, BMI, and blood glucose and the secondary outcome measure of inflammatory factors, Most studies were concentrated in the middle and upper positions of the funnel plot, showing an asymmetric distribution, which suggested a high possibility of publication bias. See Fig 8.

#### Sequential analysis of the trial

**Fig 9:**
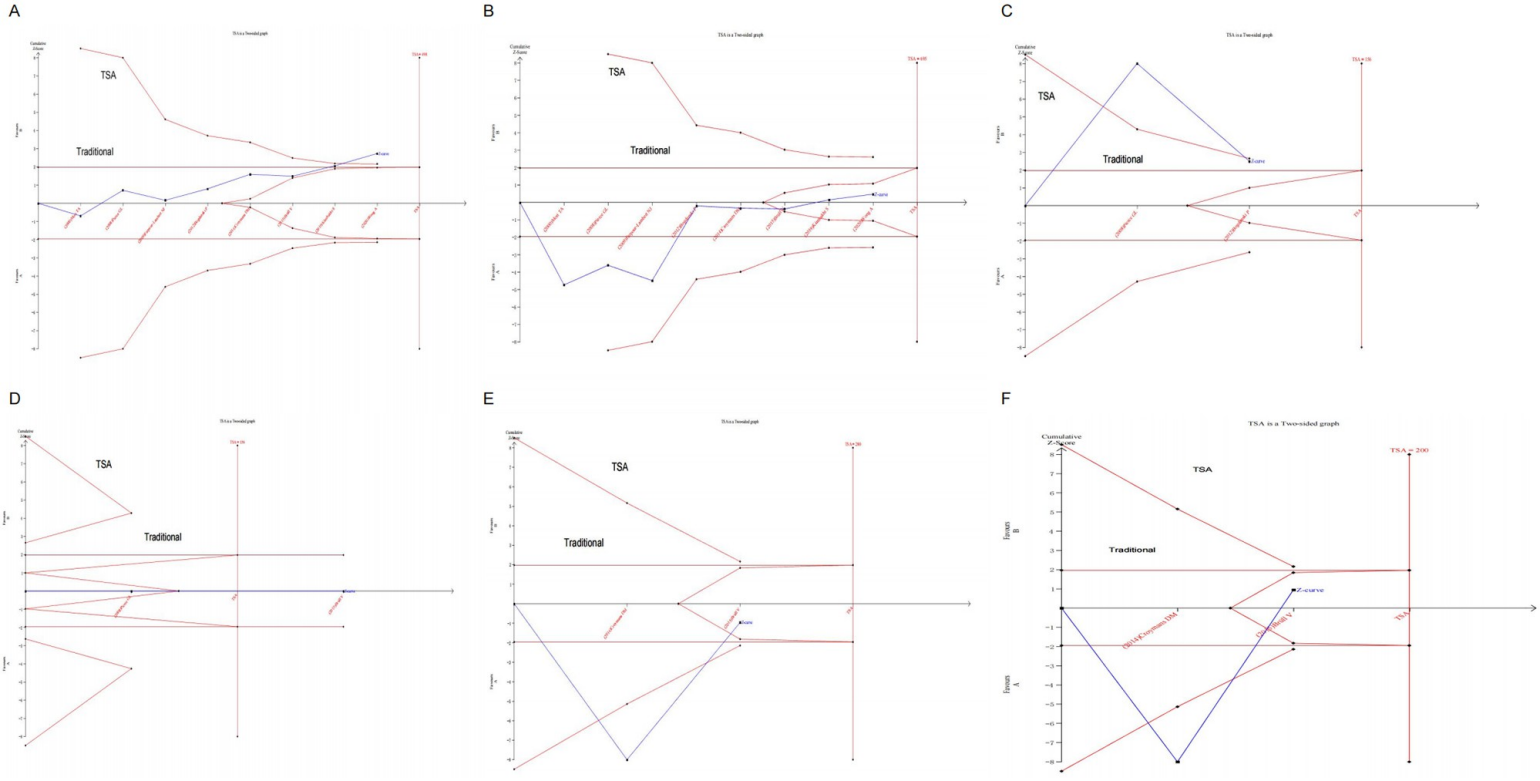
Sequential analysis of the trials (A): SBP (B): DBP (C): TNF-α (D): Endothelin-1 (E): sICAM-1 (F): sVCAM-1.

Sequential analysis was performed on systolic blood pressure, diastolic blood pressure, TNF-α, endothelin-1, sICAM-1, and sVCAM-1. Type I error was defined at 5%, and the information axis was set as the cumulative sample size, with 80%statistical. The sample size was used as the expected information value (TSA), Fig 9. The sequential analysis for systolic blood pressure, sICAM-1, and sVCAM-1 showed that the sample size had crossed the traditional cut-off value and TSA cut-off value at the time of study inclusion, and a positive conclusion was obtained in advance. However, the diastolic blood pressure reached the traditional cut-off value but did not reach the TSA cut-off value, suggesting a high risk of false-positive results. Therefore, these results require further randomized controlled trials for verification. In the sequential analysis of Endothelin-1, The Z curve did not intersect with the TSA cut-off or the conventional cut-off and did not reach the desired ideal sample size. This indicated that a large number of randomized controlled studies are required to confirm the effects of non-pharmacological treatment on obese hypertension and vascular endothelium.

#### GRADE quality evaluation

**Fig 10:**
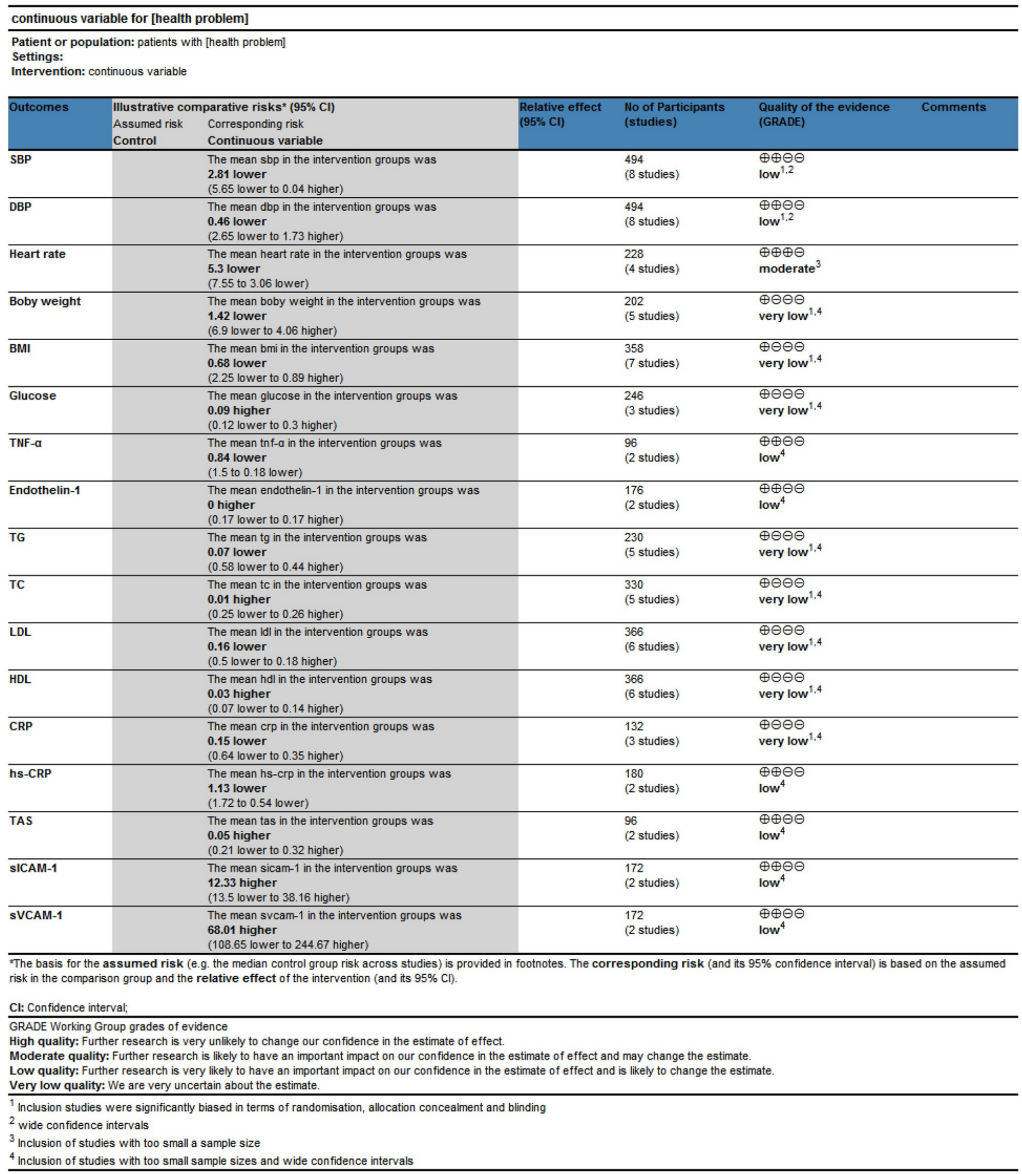
GRADE levels of evidence.

The GRADE system was used to evaluate the evidence level of various indicators, the risk of bias, consistency of results, indirectness, accuracy, and publication bias. The results showed that outcome measures of the effect of non-drug quality on hypertensive vascular endothelium in obese patients which 1 intermediate, 8 low and 8 very low grade. See Fig 10.

## Discussion

Both American and European guidelines suggest dietary interventions to lower blood pressure and suggest that dietary modification is one of the cornerstones to improving arterial hypertension. Improving lifestyle is also a mainstay of cardiovascular disease prevention [21, 22]. The literature included in this study showed that non-drug treatment could improve blood pressure, heart rate, weight, BMI, blood glucose, inflammatory factors, and vascular endothelial markers. However, due to obese hypertensive patients often having multiple diseases, it is still necessary to investigate the therapeutic effect according to the specific circumstances of the patients. A total of 8 pieces of literature were included in this study. The meta-analysis showed that non-drug treatment is particularly effective in reducing systolic blood pressure, diastolic blood pressure, heart rate, weight, BMI, blood glucose, and other indicators in obese hypertensive patients. SICAM-1 and sVCAM-1 were also improved, while no significant difference in TNF-α and Endothelin-1 was observed between the two groups. The latter may be due to the small sample size of subjects, as the sequential analysis of the trial also suggested the need for sample size validation. The inflammatory factors all showed improvement after non-drug treatment. Nevertheless, the funnel plot suggested publication bias, which may be related to the small sample size effect, Trial sequential analysis showed that non-drug treatment had conclusive evidence in improving sICAM-1 and sVCAM-1 and reducing blood pressure.

Obesity is an independent risk factor for cardiovascular disease, and numerous studies have shown that body weight plays a crucial role in blood pressure [20]. Excessive adipose tissue distribution affects hormones levels and causes corresponding inflammatory and endothelial changes. 60% of obese hypertensive patients may be attributed to increased fat stores. Furthermore, NHANES data indicated a prevalence of 42.5% for hypertension [23, 24] in obese individuals with a BMI<30 kg/m2. Studies have also demonstrated a positive correlation between BMI and the risk of hypertension, with a relatively high prevalence of hypertension in people with high BMI [25]. Obesity can lead to increased arterial stiffness and decreased vascular wall compliance [26] through the release of free fatty acids from the systemic circulation, insulin resistance, and hyperinsulinemia. The vascular endothelium dynamically maintains vascular tension, mediating angiogenesis, hemostasis, anti-oxidation, and anti-thrombosis. Endothelial dysfunction is the main pathological manifestation of cardiovascular disease, metabolic disease, and emerging infectious diseases. The release of vasodilators and the increase of vasoconstrictors are important markers of endothelial dysfunction. The mechanisms by which endothelial dysfunction leads to the development of hypertension may involve: (1) increased aortic stiffness; (2) altered vascular tension; (3) increased oxidative and nitrosative stress; (4) increased inflammatory response; (5) increased endothelin secretion [10], and so on. In the 6 included studies, HDL, LDL, CRP, hs-CRP, TNF-α, endothelin-1, FBF, and other indicators reflected varying degrees of vascular endothelial dysfunction and changes in vascular structure.

The direct or indirect treatment cost of hypertension was 46.4 billion yuan in 2011 and is expected to increase sixfold by 2030, highlighting the importance of low-cost non-drug treatment [27, 28]. Studies have shown that exercise, improvement of dietary habits, maintenance of good mood, and reduction of alcohol consumption can improve the vascular endothelium [10]. Non-pharmacological treatments have gained popularity and have been applied in various fields [29–31]. However, non-drug treatment has poor efficacy for hypertension patients with multiple diseases.

Limitations of the study: (1) A small number of studies was included and were limited to only those published in English, which overlooked other pieces of literature; (2) The age range of subjects was large but had a small sample size. In addition, publication bias was detected, requiring large, multicenter, high-quality clinical randomized controlled trials to verify the clinical efficacy. Therefore, further research is needed to apply the treatments in clinical practice; (3) Waist circumference, hip circumference, and waist-to-hip ratio have a more accurate prognostic predictive value than BMI and should be accurately measured in obese patients. (4) Only life-style and dietary supplements were analyzed as non-pharmacological treatments, while vegetable consumption, low-fat dairy products, and non-nutritional supplements were overlooked. (5) Relatively few studies have been performed on TNF-α, endothelin-1 and other endothelium-related indicators of hypertension. The efficacy of the non-pharmacological treatments on the vascular endothelium of obese hypertensive patients could not be evaluated accurately.

## Conclusions

The literature included in this study showed that non-pharmacological treatments based on life-style and dietary supplements can improve blood pressure, heart rate, weight, BMI, blood glucose and related indicators such as inflammatory factors. Due to the small sample size of the study, it was relatively ineffective in improving the endothelium, but it gives us more caution, especially in modifying blood pressure by improving life-styles, which is more desirable, and it is worth promoting and benefiting more people initially by modifying their life-styles rather than medication. Since obese hypertensive patients often have multiple morbidities, treatment plans should be tailored on a patient-by-patient basis.

## Supporting information

S1 Table Search string for PubMed, Embasa, and Cochrane Library databases. (DOCX)

S1 Checklist Reporting items for systematic review and meta-analysis (PRISMA) 2020 statement guideline. (DOCX)

## References

[1] Collaborators GBDRF. Global, regional, and national comparative risk assessment of 84 behavioral, environmental and occupational, and metabolic risks or clusters of risks for 195 countries and territories, 1990–2017: a systematic analysis for the Global Burden of Disease Study 2017. Lancet. 2018;392(10159):1923–94. Epub 2018/11/30. doi: 10.1016/S0140-6736(18)32225-6 . 30496105 PMC6227755

[2] Philip R, Beaney T, Appelbaum N, Gonzalvez CR, Koldeweij C, Golestaneh AK, et al. Variation in hypertension clinical practice guidelines: a global comparison. BMC Med. 2021;19(1):117. Epub 2021/05/13. doi: 10.1186/s12916-021-01963-0 . 33975593 PMC8114719

[3] Smith KB, Smith MS. Obesity Statistics. Prim Care. 2016;43(1):121–35, ix. Epub 2016/02/21. doi: 10.1016/j.pop.2015.10.001 . 26896205

[4] DeMarco VG, Aroor AR, Sowers JR. The pathophysiology of hypertension in patients with obesity. Nat Rev Endocrinol. 2014;10(6):364–76. Epub 2014/04/16. doi: 10.1038/nrendo.2014.44 . 24732974 PMC4308954

[5] Chrysant SG. Pathophysiology and treatment of obesity-related hypertension. J Clin Hypertens (Greenwich). 2019;21(5):555–9. Epub 2019/03/25. doi: 10.1111/jch.13518 . 30907058 PMC8030569

[6] Nugroho P, Andrew H, Kohar K, Noor CA, Sutranto AL. Comparison between the world health organization (WHO) and international society of hypertension (ISH) guidelines for hypertension. Ann Med. 2022;54(1):837–45. Epub 2022/03/17. doi: 10.1080/07853890.2022.2044510 . 35291891 PMC8933011

[7] Hall ME, Cohen JB, Ard JD, Egan BM, Hall JE, Lavie CJ, et al. Weight-Loss Strategies for Prevention and Treatment of Hypertension: A Scientific Statement From the American Heart Association. Hypertension. 2021;78(5):e38–e50. Epub 2021/09/21. doi: 10.1161/HYP.0000000000000202 . 34538096

[8] Borghi C, Tsioufis K, Agabiti-Rosei E, Burnier M, Cicero AFG, Clement D, et al. Nutraceuticals and blood pressure control: a European Society of Hypertension position document. J Hypertens. 2020;38(5):799–812. Epub 2020/01/25. doi: 10.1097/HJH.0000000000002353 . 31977574

[9] Cicero AFG, Grassi D, Tocci G, Galletti F, Borghi C, Ferri C. Nutrients and Nutraceuticals for the Management of High Normal Blood Pressure: An Evidence-Based Consensus Document. High Blood Press Cardiovasc Prev. 2019;26(1):9–25. Epub 2019/01/24. doi: 10.1007/s40292-018-0296-6 . 30671873

[10] Xu S, Ilyas I, Little PJ, Li H, Kamato D, Zheng X, et al. Endothelial Dysfunction in Atherosclerotic Cardiovascular Diseases and Beyond: From Mechanism to Pharmacotherapies. Pharmacol Rev. 2021;73(3):924–67. Epub 2021/06/06. doi: 10.1124/pharmrev.120.000096 . 34088867

[11] Oliveira GF, Marin TC, Forjaz CLM, Brito LC. Exercise Training and Endothelial Function in Hypertension: Effects of Aerobic and Resistance Training. Arq Bras Cardiol. 2021;116(5):948–9. Epub 2021/05/20. doi: 10.36660/abc.20210111 . 34008819 PMC8121457

[12] Moher D, Shamseer L, Clarke M, Ghersi D, Liberati A, Petticrew M, et al. Preferred reporting items for systematic review and meta-analysis protocols (PRISMA-P) 2015 statement. Syst Rev. 2015;4:1. Epub 2015/01/03. doi: 10.1186/2046-4053-4-1 . 25554246 PMC4320440

[13] Bogdanski P, Suliburska J, Szulinska M, Stepien M, Pupek-Musialik D, Jablecka A. Green tea extract reduces blood pressure, inflammatory biomarkers, and oxidative stress and improves parameters associated with insulin resistance in obese, hypertensive patients. Nutr Res. 2012;32(6):421–7. Epub 2012/07/04. doi: 10.1016/j.nutres.2012.05.007 . 22749178

[14] Brull V, Burak C, Stoffel-Wagner B, Wolffram S, Nickenig G, Muller C, et al. Effects of a quercetin-rich onion skin extract on 24 h ambulatory blood pressure and endothelial function in overweight-to-obese patients with (pre-)hypertension: a randomized double-blinded placebo-controlled cross-over trial. Br J Nutr. 2015;114(8):1263–77. Epub 2015/09/04. doi: 10.1017/S0007114515002950 . 26328470 PMC4594049

[15] Mori TA, Watts GF, Burke V, Hilme E, Puddey IB, Beilin LJ. Differential effects of eicosapentaenoic acid and docosahexaenoic acid on vascular reactivity of the forearm microcirculation in hyperlipidemic, overweight men. Circulation. 2000;102(11):1264–9. Epub 2000/09/12. doi: 10.1161/01.cir.102.11.1264 . 10982541

[16] Pierce GL, Beske SD, Lawson BR, Southall KL, Benay FJ, Donato AJ, et al. Weight loss alone improves conduit and resistance artery endothelial function in young and older overweight/obese adults. Hypertension. 2008;52(1):72–9. Epub 2008/05/28. doi: 10.1161/HYPERTENSIONAHA.108.111427 . 18504322 PMC2913284

[17] Farpour-Lambert NJ, Aggoun Y, Marchand LM, Martin XE, Herrmann FR, Beghetti M. Physical activity reduces systemic blood pressure and improves early markers of atherosclerosis in pre-pubertal obese children. J Am Coll Cardiol. 2009;54(25):2396–406. Epub 2010/01/20. doi: 10.1016/j.jacc.2009.08.030 . 20082930

[18] Croymans DM, Krell SL, Oh CS, Katiraie M, Lam CY, Harris RA, et al. Effects of resistance training on central blood pressure in obese young men. J Hum Hypertens. 2014;28(3):157–64. Epub 2013/09/06. doi: 10.1038/jhh.2013.81 . 24005959 PMC4119468

[19] Wong A, Figueroa A, Fischer SM, Bagheri R, Park SY. The Effects of Mat Pilates Training on Vascular Function and Body Fatness in Obese Young Women With Elevated Blood Pressure. Am J Hypertens. 2020;33(6):563–9. Epub 2020/04/03. doi: 10.1093/ajh/hpaa026 . 32236522

[20] Kianbakht S, Hashem-Dabaghian F. Antihypertensive efficacy and safety of Vaccinium arctostaphylos berry extract in overweight/obese hypertensive patients: A randomized, double-blind and placebo-controlled clinical trial. Complement Ther Med. 2019;44:296–300. Epub 2019/05/28. doi: 10.1016/j.ctim.2019.05.010 . 31126570

[21] Cicero AFG, Veronesi M, Fogacci F. Dietary Intervention to Improve Blood Pressure Control: Beyond Salt Restriction. High Blood Press Cardiovasc Prev. 2021;28(6):547–53. Epub 2021/09/18. doi: 10.1007/s40292-021-00474-6 . 34533781 PMC8590666

[22] Strilchuk L, Cincione RI, Fogacci F, Cicero AFG. Dietary interventions in blood pressure lowering: current evidence in 2020. Kardiol Pol. 2020;78(7–8):659–66. Epub 2020/07/08. doi: 10.33963/KP.15468 . 32631027

[23] Landsberg L, Aronne LJ, Beilin LJ, Burke V, Igel LI, Lloyd-Jones D, et al. Obesity-related hypertension: pathogenesis, cardiovascular risk, and treatment: a position paper of The Obesity Society and the American Society of Hypertension. J Clin Hypertens (Greenwich). 2013;15(1):14–33. Epub 2013/01/04. doi: 10.1111/jch.12049 . 23282121 PMC8108268

[24] Seravalle G, Grassi G. Corrigendum to "Obesity and hypertension" [Pharmacol. Res. 122 (2017) 1–7]. Pharmacol Res. 2017;124:156. Epub 2017/08/06. doi: 10.1016/j.phrs.2017.07.018 . 28778374

[25] Zhou W, Shi Y, Li YQ, Ping Z, Wang C, Liu X, et al. Body mass index, abdominal fatness, and hypertension incidence: a dose-response meta-analysis of prospective studies. J Hum Hypertens. 2018;32(5):321–33. Epub 2018/03/28. doi: 10.1038/s41371-018-0046-1 . 29581553

[26] Fantin F, Giani A, Zoico E, Rossi AP, Mazzali G, Zamboni M. Weight Loss and Hypertension in Obese Subjects. Nutrients. 2019;11(7). Epub 2019/07/25. doi: 10.3390/nu11071667 . 31330870 PMC6682923

[27] Sabbahi A, Arena R, Elokda A, Phillips SA. Exercise and Hypertension: Uncovering the Mechanisms of Vascular Control. Prog Cardiovasc Dis. 2016;59(3):226–34. Epub 2016/10/27. doi: 10.1016/j.pcad.2016.09.006 . 27697533

[28] Carey RM, Whelton PK, Committee AAHGW. Prevention, Detection, Evaluation, and Management of High Blood Pressure in Adults: Synopsis of the 2017 American College of Cardiology/American Heart Association Hypertension Guideline. Ann Intern Med. 2018;168(5):351–8. Epub 2018/01/23. doi: 10.7326/M17-3203 . 29357392

[29] Mahmood S, Shah KU, Khan TM, Nawaz S, Rashid H, Baqar SWA, et al. Non-pharmacological management of hypertension: in the light of current research. Ir J Med Sci. 2019;188(2):437–52. Epub 2018/08/24. doi: 10.1007/s11845-018-1889-8 . 30136222

[30] Abraha I, Rimland JM, Trotta FM, Dell’Aquila G, Cruz-Jentoft A, Petrovic M, et al. Systematic review of systematic reviews of non-pharmacological interventions to treat behavioural disturbances in older patients with dementia. The SENATOR-OnTop series. BMJ Open. 2017;7(3):e012759. Epub 2017/03/18. doi: 10.1136/bmjopen-2016-012759 . 28302633 PMC5372076

[31] Fu J, Liu Y, Zhang L, Zhou L, Li D, Quan H, et al. Nonpharmacologic Interventions for Reducing Blood Pressure in Adults With Prehypertension to Established Hypertension. J Am Heart Assoc. 2020;9(19):e016804. Epub 2020/09/26. doi: 10.1161/JAHA.120.016804 . 32975166 PMC7792371

