# Supplementary material for: Metabolic status and vascular endothelial structure in obese hypertensive patients treated with non-pharmacological therapies: A systematic review and meta-analysis
Source: PLoS One. 2022 Dec 30;17(12):e0279582. doi: 10.1371/journal.pone.0279582 (PMC9803191; doi:10.1371/journal.pone.0279582)
Supplement: S1 Table — (DOCX) [file pone.0279582.s001.docx]

Table: Search string for PubMed、Embase,and Cochrane Library databases

| Databases | Search | Search terms |
| --- | --- | --- |
| **PubMed** | #1 | "Hypertension"[Mesh] |
|  | #2 | (((Blood Pressure, High[Title/Abstract]) OR (Blood Pressures, High[Title/Abstract])) OR (High Blood Pressure[Title/Abstract])) OR (High Blood Pressures[Title/Abstract]) |
|  | #3 | ("Hypertension"[Mesh]) OR ((((Blood Pressure, High[Title/Abstract]) OR (Blood Pressures, High[Title/Abstract])) OR (High Blood Pressure[Title/Abstract])) OR (High Blood Pressures[Title/Abstract])) |
|  | #4 | "Weight Loss"[Majr] |
|  | #5 | ((((((Loss, Weight[Title/Abstract]) OR (Losses, Weight[Title/Abstract])) OR (Weight Losses[Title/Abstract])) OR (Weight Reduction[Title/Abstract])) OR (Reduction, Weight[Title/Abstract])) OR (Reductions, Weight[Title/Abstract])) OR (Weight Reductions[Title/Abstract]) |
|  | #6 | ("Weight Loss"[Majr]) OR (((((((Loss, Weight[Title/Abstract]) OR (Losses, Weight[Title/Abstract])) OR (Weight Losses[Title/Abstract])) OR (Weight Reduction[Title/Abstract])) OR (Reduction, Weight[Title/Abstract])) OR (Reductions, Weight[Title/Abstract])) OR (Weight Reductions[Title/Abstract])) |
|  | #7 | randomized controlled trial[Publication Type] OR randomized[Title/Abstract] OR placebo[Title/Abstract] |
|  | #8 | ((("Hypertension"[Mesh]) OR ((((Blood Pressure, High[Title/Abstract]) OR (Blood Pressures, High[Title/Abstract])) OR (High Blood Pressure[Title/Abstract])) OR (High Blood Pressures[Title/Abstract]))) AND (("Weight Loss"[Majr]) OR (((((((Loss, Weight[Title/Abstract]) OR (Losses, Weight[Title/Abstract])) OR (Weight Losses[Title/Abstract])) OR (Weight Reduction[Title/Abstract])) OR (Reduction, Weight[Title/Abstract])) OR (Reductions, Weight[Title/Abstract])) OR (Weight Reductions[Title/Abstract])))) AND (randomized controlled trial[Publication Type] OR randomized[Title/Abstract] OR placebo[Title/Abstract]) |
| **Embase, and Cochrane Library databases** | #1 | ("Weight Loss"[Majr]) OR (((((((Loss, Weight[Title/Abstract]) OR (Losses, Weight[Title/Abstract])) OR (Weight Losses[Title/Abstract])) OR (Weight Reduction[Title/Abstract])) OR (Reduction, Weight[Title/Abstract])) OR (Reductions, Weight[Title/Abstract])) OR (Weight Reductions[Title/Abstract])) |
