# Supplementary material for: Metabolic status and vascular endothelial structure in obese hypertensive patients treated with non-pharmacological therapies: A systematic review and meta-analysis
Source: PLoS One. 2022 Dec 30;17(12):e0279582. doi: 10.1371/journal.pone.0279582 (PMC9803191; doi:10.1371/journal.pone.0279582)
Supplement: Attachment — Submitted filename: Response to Reviewers.docx [file pone.0279582.s003.docx]

Dear reviewer:

Thank you for your comments concerning our manuscript entitled “Metabolic status and vascular endothelial structure in obese hypertensive patients treated with non-pharmacological therapies: a systematic review and meta-analysis” (Manuscript ID:PONE-D-22-16167). Those constructive comments are all valuable and very helpful for revising and improving our paper, as well as the important guiding significance to our researches. We have studied comments carefully and have made correction which we hope meet with approval. And highlighting function is used for all corrections in the maniscript. For accurate answer, we separated the report into 12 comments, and replied each comment respectively.

**Commment 1:** ACADEMIC EDITOR: Professional proofreading in English is required.The excess of acronyms in the summary left the result incomprehensible. Better to prioritize the most important results and keep them in line with the degree of uncertainty.

**Authors’ Response:** We tried our best to improve the manuscript and made some changes to the manuscript. These changes will not influence the content and framework of the paper. And here we did not list the changes but marked in red in the revised paper, with emphasis in lines 26 to 31. We appreciate for Editors/Reviewers’ warm work earnestly and hope that the correction will meet with approval and to regularise and amend the abstract.

**Commment 2:** Table 1 is mind-boggling. I suggest putting it as supplementary material.It is necessary to describe any methods used to assess the certainty (or confidence) in the body of evidence for an outcome. In the results, follow the PRISMA sequence: Study selection; Study characteristics; Risk of bias in studies; Results of individual studies; Synthesis results; biased reports; Evidence certainty.

**Authors’ Response:** Thank you very much for your valuable suggestions. It has been included as supplementary material. In the results, the order of PRISMA has been followed.

Table: Search string for PubMed、Embase,and Cochrane Library databases

| Databases | Search | Search terms |
| --- | --- | --- |
| **PubMed** | #1 | "Hypertension"[Mesh] |
|  | #2 | (((Blood Pressure, High[Title/Abstract]) OR (Blood Pressures, High[Title/Abstract])) OR (High Blood Pressure[Title/Abstract])) OR (High Blood Pressures[Title/Abstract]) |
|  | #3 | ("Hypertension"[Mesh]) OR ((((Blood Pressure, High[Title/Abstract]) OR (Blood Pressures, High[Title/Abstract])) OR (High Blood Pressure[Title/Abstract])) OR (High Blood Pressures[Title/Abstract])) |
|  | #4 | "Weight Loss"[Majr] |
|  | #5 | ((((((Loss, Weight[Title/Abstract]) OR (Losses, Weight[Title/Abstract])) OR (Weight Losses[Title/Abstract])) OR (Weight Reduction[Title/Abstract])) OR (Reduction, Weight[Title/Abstract])) OR (Reductions, Weight[Title/Abstract])) OR (Weight Reductions[Title/Abstract]) |
|  | #6 | ("Weight Loss"[Majr]) OR (((((((Loss, Weight[Title/Abstract]) OR (Losses, Weight[Title/Abstract])) OR (Weight Losses[Title/Abstract])) OR (Weight Reduction[Title/Abstract])) OR (Reduction, Weight[Title/Abstract])) OR (Reductions, Weight[Title/Abstract])) OR (Weight Reductions[Title/Abstract])) |
|  | #7 | randomized controlled trial[Publication Type] OR randomized[Title/Abstract] OR placebo[Title/Abstract] |
|  | #8 | ((("Hypertension"[Mesh]) OR ((((Blood Pressure, High[Title/Abstract]) OR (Blood Pressures, High[Title/Abstract])) OR (High Blood Pressure[Title/Abstract])) OR (High Blood Pressures[Title/Abstract]))) AND (("Weight Loss"[Majr]) OR (((((((Loss, Weight[Title/Abstract]) OR (Losses, Weight[Title/Abstract])) OR (Weight Losses[Title/Abstract])) OR (Weight Reduction[Title/Abstract])) OR (Reduction, Weight[Title/Abstract])) OR (Reductions, Weight[Title/Abstract])) OR (Weight Reductions[Title/Abstract])))) AND (randomized controlled trial[Publication Type] OR randomized[Title/Abstract] OR placebo[Title/Abstract]) |
| **Embase, and Cochrane Library databases** | #1 | ("Weight Loss"[Majr]) OR (((((((Loss, Weight[Title/Abstract]) OR (Losses, Weight[Title/Abstract])) OR (Weight Losses[Title/Abstract])) OR (Weight Reduction[Title/Abstract])) OR (Reduction, Weight[Title/Abstract])) OR (Reductions, Weight[Title/Abstract])) OR (Weight Reductions[Title/Abstract])) |

**Commment 3:** Table 2 is incomprehensible, I suggest redoing it.Meta-analyses need to be redone: plot by outcome and create subgroups for the interventions included in the analysis.

**Authors’ Response:** We think this is an excellent suggestion. The tables and Meta-analysis results have then been redrawn.

Basic information of the included literature Table

| **Author,Year** | **Cases** | | **Age (years)** | | **Treatment** | | **Intervention period /month** | **Outcome indicator** |
| --- | --- | --- | --- | --- | --- | --- | --- | --- |
|  | **T** | **C** | **T** | **C** | **T** | **C** |  |  |
| Mori TA,2000 [15] | 26 | 14 | 50.8±2.0 | 50.6±1.4 | EPA/DHA | Placebo | 1.5 | SBP,DBP,Bodyweight,BMI,TNF-α,LDL, HDL,TC,TG,Glucose,CRP,TAS |
| Pierce GL,2008 [16] | 26 | 14 | 49.2±2.5 | 40.8±3.3 | Weight Loss | Untreated | 4 | SBP,DBP,Heart rate, Body weight,BMI,ET-1,TNF-α,LDL,HDL,TC,TG,Glucose |
| Farpour-Lambert NJ, 2009[17] | 22 | 22 | 9.1±1.4 | 8.8±1.6 | Obese Exercise | Untreated | 3 | SBP,DBP, Body weight,BMI, LDL,HDL,TC,TG,FBF |
| Bogdanski P,2012 [13] | 28 | 28 | 49.2±8.8 | 51.5±7.4 | Green tea extract | Placebo | 3 | SBP,DBP, Body weight,BMI, LDL,HDL,TC,TG, Glucose ,FBF |
| Croymans DM,2014 [18] | 28 | 8 | 22.0 | 21.5 | resistance training | Untreated | 3 | SBP,DBP,Heart rate,Body weight,BMI,sICAM-1,sVCAM-1,LDL,HDL,TG,CRP |
| Brüll V,2015 [14] | 68 | 68 | 7.4±10.5 | 59.6±4.3 | Quercetin | Placebo | 1.5 | SBP,DBP,Heart rate,Body weight,BMI,sICAM-1,sVCAM-1, LDL,HDL,TG,CRP |
| Kianbakht S,2019[20] | 50 | 50 | 57±6 | 61±8 | V. arctostaphylos berry extract | Placebo | 3 | SBP,DBP, Heart rate, sICAM-1,sVCAM-1,ET-1,LDL,HDL,TG,CRP |
| Wong A,2020[19] | 14 | 14 | 22±1 | 23±1 | Placebo | Untreated | 3 | SBP,DBP,BMI |

Note：T: The treatment group； C: Control group；

Remapping has been carried out depending on the intervention, there are two types of lifestyle and dietary supplements.

Fig3. Forest plot of primary outcome indicators(I)

(A) SBP Forest plot of primary outcome indicators (B) DBP Forest plot of primary outcome indicators (C) Heart rate Forest plot of primary outcome indicators

Fig 4.Forest plot of primary outcome indicators(II)

(A) Boby weight Forest plot of primary outcome indicators (B) BMI Forest plot of primary outcome indicators (C) Glucose Forest plot of primary outcome indicators

Fig 5.Forest plot of primary outcome indicators(III)

(A) TNF-α Forest plot of primary outcome indicators (B) Endothelin-1 Forest plot of primary outcome indicators (C) sICAM-1Forest plot of primary outcome indicators(D) sVCAM-1 Forest plot of primary outcome indicators

Fig 6.Forest map secondary outcome indicators

(A) TG Forest map secondary outcome indicators (B) TC Forest map secondary outcome indicators (C) HDL Forest map secondary outcome indicators (D) LDL Forest map secondary outcome indicators

Fig 7.Forest map secondary outcome indicators

(A) CRP Forest map secondary outcome indicators (B) hs-CRP Forest map secondary outcome indicators (C) TAS Forest map secondary outcome indicators

**Commment 4:** GRADE Table 3 is incomprehensible and incomplete: it is best to redo it using GRADE Pro.

**Authors’ Response:** Your suggestion really means a lot to us，already redrawn with GRADE Pro.

Fig 10.GRADE levels of Evidence

**Commment 5:** The authors mixed the effect of totally different interventions (life-style and dietary supplements) trying to drive a common conclusion. This makes not sense from a clinical point of view and the conclusion are supported by statistical analysis, but are not usefult.

**Authors’ Response:**  First of all, thank you very much for your valuable comments, which point out the shortcomings of this paper. We have now compared the observed indicators by lifestyle and dietary supplements in a subgroup analysis, and it is clear from the indicators analysed that the effect of lifestyle changes in lowering blood pressure and improving the endothelium is more pronounced. A European Society of Hypertension position document has clearly stated the status of Nutraceuticals and blood pressure control, the aim of this paper is to make more people aware of the benefits of non-pharmacological treatments and to promote them so that more people can benefit.

**Commment 6:** Figure 1: The authors should explain what do they mean by "study design is not rigorous". The authors should also assess for the presence of publication bias in the analysis by performing the Egger and Begg's tests.

**Authors’ Response:** Firstly, there are limitations in the six papers, all of which relate to the endothelium of non-pharmacological obese hypertensive patients; The first article: no randomised sequential analysis, no blinding and too small a sample size; The second article: no blinded evaluation of the results and relatively limited participation in the study; The third article: no hidden allocation of participants and no comparison of the treatment of the trial and control groups. In the fourth article, dietary therapy was used for both the test and control groups, and no comparison was made between the normal group; In the fifth and sixth articles, no control observations were made, especially in the sixth article, which used different groups of thin and fat people, with a large margin of error, and no post-treatment observation was made on the same person. The following six articles are DOI:DOI: [10.1016/s0895-7061(01)02322-6](https://doi.org/10.1016/s0895-7061(01)02322-6)、DOI: [10.1080/07315724.2016.1194236](https://doi.org/10.1080/07315724.2016.1194236)、DOI: [10.1038/jhh.2009.58](https://doi.org/10.1038/jhh.2009.58) 、DOI: [10.3109/08037051.2014.976979](https://doi.org/10.3109/08037051.2014.976979)、

DOI: [10.1016/j.numecd.2010.03.003](https://doi.org/10.1016/j.numecd.2010.03.003) 、DOI: [10.1016/s0895-7061(01)02191-4](https://doi.org/10.1016/s0895-7061(01)02191-4)

The Egger and Begg's tests were not biased because the third, fifth, and sixth articles were not subjected to controlled observations.

The following are the first, second and fourth Egger and Begg's tests

由于Begg's tests P=1; Egger tests P=0.29, not statistically significant when p>0.05.

**Commment 7:** The limitations of the study should be further and more deeply discussed.

**Authors’ Response:** Only life-styl and dietary supplements were analyzed for non-pharmacological treatment, and there was a lack of observation and analysis for vegetables, low-fat dairy products, and non-nutritional supplements.There are relatively few studies on TNF-α, Endothelin-1 and other endothelium-related indicators of hypertension, and the effects are not good enough to further explore the extent to which their non-pharmacological effects on the vascular endothelium of obese hypertensive patients.

**Commment 8:** The authors should highly consider to include in their manuscript the references doi: 10.1007/s40292-021-00474-6, doi: 10.33963/KP.15468, doi: 10.1097/HJH.0000000000002353 and doi: 10.1007/s40292-018-0296-6

**Authors’ Response:** These papers are of great value to this paper and have been added to this paper, Lines 54-56 and 264-267, respectively, with references 8, 9 and 21, 22.

**Commment 9:** Please ensure that your manuscript meets PLOS ONE's style requirements, including those for file naming.

**Authors’ Response:** Already modified according to the PLOS ONE's style requirements.

**Commment 10:** Thank you for stating the following financial disclosure: "NO".At this time, please address the following queries:a) Please clarify the sources of funding (financial or material support) for your study. List the grants or organizations that supported your study, including funding received from your institution. b) State what role the funders took in the study. If the funders had no role in your study, please state: “The funders had no role in study design, data collection and analysis, decision to publish, or preparation of the manuscript.”c) If any authors received a salary from any of your funders, please state which authors and which funders.d) If you did not receive any funding for this study, please state: “The authors received no specific funding for this work.Please include your amended statements within your cover letter; we will change the online submission form on your behalf.

**Authors’ Response:**Funding:Jinan Science and Technology Bureau Clinical Medical Science and Technology Innovation Program (No. 202134024), YC was involved in the conception and design of the study and provided scientific oversight.

**Commment 11:**Thank you for stating the following in your Competing Interests section: "NO authors have competing interestsEnter: The authors have declared that no competing interests exist."Please complete your Competing Interests on the online submission form to state any Competing Interests. If you have no competing interests, please state "The authors have declared that no competing interests exist.", as detailed online in our guide for authors at http://journals.plos.org/plosone/s/submit-now.This information should be included in your cover letter; we will change the online submission form on your behalf.

**Authors’ Response:** "The authors have declared that no competing interests exist."

**Commment 12:**Please upload a copy of Figure 9, to which you refer in your text on page 13. If the figure is no longer to be included as part of the submission please remove all reference to it within the text.

**Authors’ Response:**Fig1.:SBP Fig2.:DBP Fig3.:TNF-αFig4.:Endothelin-1 Fig5.: sICAM-1 Fig6.:sVCAM-1

Fig1.

Fig2.

Fig3. Fig4.

Fig5.

Fig6.
