# Supplementary material for: Metabolic status and vascular endothelial structure in obese hypertensive patients treated with non-pharmacological therapies: A systematic review and meta-analysis
Source: PLoS One. 2022 Dec 30;17(12):e0279582. doi: 10.1371/journal.pone.0279582 (PMC9803191; doi:10.1371/journal.pone.0279582)
Supplement: Peer review history

# Peer review: Decision Letter 0

**DOI:** 10.1371/journal.pone.0279582.r001

**Authors:** Marcus Tolentino Silva

27 Oct 2022

PONE-D-22-16167Metabolic status and vascular endothelial structure in obese hypertensive patients treated with non-pharmacological therapies: a systematic review and meta-analysisPLOS ONE

Dear Dr. CHEN,

Thank you for submitting your manuscript to PLOS ONE. After careful consideration, we feel that it has merit but does not fully meet PLOS ONE’s publication criteria as it currently stands. Therefore, we invite you to submit a revised version of the manuscript that addresses the points raised during the review process.

==============================

ACADEMIC EDITOR: Professional proofreading in English is required.

The excess of acronyms in the summary left the result incomprehensible. Better to prioritize the most important results and keep them in line with the degree of uncertainty.

Table 1 is mind-boggling. I suggest putting it as supplementary material.

It is necessary to describe any methods used to assess the certainty (or confidence) in the body of evidence for an outcome.

In the results, follow the PRISMA sequence: Study selection; Study characteristics; Risk of bias in studies; Results of individual studies; Synthesis results; biased reports; Evidence certainty.

Table 2 is incomprehensible, I suggest redoing it.

Meta-analyses need to be redone: plot by outcome and create subgroups for the interventions included in the analysis.

GRADE Table 3 is incomprehensible and incomplete: it is best to redo it using GRADE Pro.

==============================

Please submit your revised manuscript by Dec 11 2022 11:59PM. If you will need more time than this to complete your revisions, please reply to this message or contact the journal office at plosone@plos.org. When you're ready to submit your revision, log on to https://www.editorialmanager.com/pone/ and select the 'Submissions Needing Revision' folder to locate your manuscript file.

Please include the following items when submitting your revised manuscript:

- A rebuttal letter that responds to each point raised by the academic editor and reviewer(s). You should upload this letter as a separate file labeled 'Response to Reviewers'.
- A marked-up copy of your manuscript that highlights changes made to the original version. You should upload this as a separate file labeled 'Revised Manuscript with Track Changes'.
- An unmarked version of your revised paper without tracked changes. You should upload this as a separate file labeled 'Manuscript'.

If you would like to make changes to your financial disclosure, please include your updated statement in your cover letter. Guidelines for resubmitting your figure files are available below the reviewer comments at the end of this letter.

If applicable, we recommend that you deposit your laboratory protocols in protocols.io to enhance the reproducibility of your results. Protocols.io assigns your protocol its own identifier (DOI) so that it can be cited independently in the future. For instructions see: https://journals.plos.org/plosone/s/submission-guidelines#loc-laboratory-protocols. Additionally, PLOS ONE offers an option for publishing peer-reviewed Lab Protocol articles, which describe protocols hosted on protocols.io. Read more information on sharing protocols at https://plos.org/protocols?utm_medium=editorial-email&utm_source=authorletters&utm_campaign=protocols.

We look forward to receiving your revised manuscript.

Kind regards,

Marcus Tolentino Silva

Academic Editor

PLOS ONE

Journal Requirements:

When submitting your revision, we need you to address these additional requirements.

1. Please ensure that your manuscript meets PLOS ONE's style requirements, including those for file naming. The PLOS ONE style templates can be found at 

https://journals.plos.org/plosone/s/file?id=wjVg/PLOSOne_formatting_sample_main_body.pdf and 

https://journals.plos.org/plosone/s/file?id=ba62/PLOSOne_formatting_sample_title_authors_affiliations.pdf

2. Thank you for stating the following financial disclosure: 

"NO".

At this time, please address the following queries:

a) Please clarify the sources of funding (financial or material support) for your study. List the grants or organizations that supported your study, including funding received from your institution. 

b) State what role the funders took in the study. If the funders had no role in your study, please state: “The funders had no role in study design, data collection and analysis, decision to publish, or preparation of the manuscript.”

c) If any authors received a salary from any of your funders, please state which authors and which funders.

d) If you did not receive any funding for this study, please state: “The authors received no specific funding for this work.

Please include your amended statements within your cover letter; we will change the online submission form on your behalf.

3. Thank you for stating the following in your Competing Interests section:  

"NO authors have competing interests

Enter: The authors have declared that no competing interests exist."

Please complete your Competing Interests on the online submission form to state any Competing Interests. If you have no competing interests, please state "The authors have declared that no competing interests exist.", as detailed online in our guide for authors at http://journals.plos.org/plosone/s/submit-now 

 This information should be included in your cover letter; we will change the online submission form on your behalf.

4. Please upload a copy of Figure 9, to which you refer in your text on page 13. If the figure is no longer to be included as part of the submission please remove all reference to it within the text.

5. Please include captions for your Supporting Information files at the end of your manuscript, and update any in-text citations to match accordingly. Please see our Supporting Information guidelines for more information: http://journals.plos.org/plosone/s/supporting-information. 

[Note: HTML markup is below. Please do not edit.]

Reviewers' comments:

Reviewer's Responses to Questions

**Comments to the Author**

1. Is the manuscript technically sound, and do the data support the conclusions?

The manuscript must describe a technically sound piece of scientific research with data that supports the conclusions. Experiments must have been conducted rigorously, with appropriate controls, replication, and sample sizes. The conclusions must be drawn appropriately based on the data presented. 

Reviewer #1: No

Reviewer #2: Partly

**********

2. Has the statistical analysis been performed appropriately and rigorously? 

Reviewer #1: Yes

Reviewer #2: Yes

**********

3. Have the authors made all data underlying the findings in their manuscript fully available?

The PLOS Data policy requires authors to make all data underlying the findings described in their manuscript fully available without restriction, with rare exception (please refer to the Data Availability Statement in the manuscript PDF file). The data should be provided as part of the manuscript or its supporting information, or deposited to a public repository. For example, in addition to summary statistics, the data points behind means, medians and variance measures should be available. If there are restrictions on publicly sharing data—e.g. participant privacy or use of data from a third party—those must be specified.

Reviewer #1: Yes

Reviewer #2: Yes

**********

4. Is the manuscript presented in an intelligible fashion and written in standard English?

PLOS ONE does not copyedit accepted manuscripts, so the language in submitted articles must be clear, correct, and unambiguous. Any typographical or grammatical errors should be corrected at revision, so please note any specific errors here.

Reviewer #1: No

Reviewer #2: No

**********

5. Review Comments to the Author

Please use the space provided to explain your answers to the questions above. You may also include additional comments for the author, including concerns about dual publication, research ethics, or publication ethics. (Please upload your review as an attachment if it exceeds 20,000 characters)

Reviewer #1: The authors mixed the effect of totally different interventions (life-style and dietary supplements) trying to drive a common conclusion. This makes not sense from a clinical point of view and the conclusion are supported by statistical analysis, but are not usefult.

Reviewer #2: Dear Editor,

I carefully read the manuscript "Metabolic status and vascular endothelial structure in obese hypertensive patients

treated with non-pharmacological therapies: a systematic review and meta-analysis", by Chen et al.

My comments and suggestions for the authors are the following:

- English language needs to be carefully revised and improved.

- Figure 1: The authors should explain what do they mean by "study design is not rigorous".

- The authors should also assess for the presence of publication bias in the analysis by performing the Egger and Begg's tests.

- The limitations of the study should be further and more deeply discussed.

- The authors should highly consider to include in their manuscript the references doi: 10.1007/s40292-021-00474-6, doi: 10.33963/KP.15468, doi: 10.1097/HJH.0000000000002353 and doi: 10.1007/s40292-018-0296-6.

**********

6. PLOS authors have the option to publish the peer review history of their article (what does this mean?). If published, this will include your full peer review and any attached files.

If you choose “no”, your identity will remain anonymous but your review may still be made public.

**Do you want your identity to be public for this peer review?** For information about this choice, including consent withdrawal, please see our Privacy Policy.

Reviewer #1: No

Reviewer #2: No

**********

[NOTE: If reviewer comments were submitted as an attachment file, they will be attached to this email and accessible via the submission site. Please log into your account, locate the manuscript record, and check for the action link "View Attachments". If this link does not appear, there are no attachment files.]

While revising your submission, please upload your figure files to the Preflight Analysis and Conversion Engine (PACE) digital diagnostic tool, https://pacev2.apexcovantage.com/. PACE helps ensure that figures meet PLOS requirements. To use PACE, you must first register as a user. Registration is free. Then, login and navigate to the UPLOAD tab, where you will find detailed instructions on how to use the tool. If you encounter any issues or have any questions when using PACE, please email PLOS at figures@plos.org. Please note that Supporting Information files do not need this step.

# Author comment: Author response to Decision Letter 0

**DOI:** 10.1371/journal.pone.0279582.r002

23 Nov 2022

Dear reviewer:

Thank you for your comments concerning our manuscript entitled “Metabolic status and vascular endothelial structure in obese hypertensive patients treated with non-pharmacological therapies: a systematic review and meta-analysis” (Manuscript ID:PONE-D-22-16167). Those constructive comments are all valuable and very helpful for revising and improving our paper, as well as the important guiding significance to our researches. We have studied comments carefully and have made correction which we hope meet with approval. And highlighting function is used for all corrections in the maniscript. For accurate answer, we separated the report into 12 comments, and replied each comment respectively. 

Commment 1: ACADEMIC EDITOR: Professional proofreading in English is required.The excess of acronyms in the summary left the result incomprehensible. Better to prioritize the most important results and keep them in line with the degree of uncertainty. 

Authors’ Response: We tried our best to improve the manuscript and made some changes to the manuscript. These changes will not influence the content and framework of the paper. And here we did not list the changes but marked in red in the revised paper, with emphasis in lines 26 to 31. We appreciate for Editors/Reviewers’ warm work earnestly and hope that the correction will meet with approval and to regularise and amend the abstract.

Commment 2: Table 1 is mind-boggling. I suggest putting it as supplementary material.It is necessary to describe any methods used to assess the certainty (or confidence) in the body of evidence for an outcome. In the results, follow the PRISMA sequence: Study selection; Study characteristics; Risk of bias in studies; Results of individual studies; Synthesis results; biased reports; Evidence certainty.

Authors’ Response: Thank you very much for your valuable suggestions. It has been included as supplementary material. In the results, the order of PRISMA has been followed.

Table: Search string for PubMed、Embase,and Cochrane Library databases

Databases Search Search terms

PubMed #1 "Hypertension"[Mesh]

 #2 (((Blood Pressure, High[Title/Abstract]) OR (Blood Pressures, High[Title/Abstract])) OR (High Blood Pressure[Title/Abstract])) OR (High Blood Pressures[Title/Abstract])

 #3 ("Hypertension"[Mesh]) OR ((((Blood Pressure, High[Title/Abstract]) OR (Blood Pressures, High[Title/Abstract])) OR (High Blood Pressure[Title/Abstract])) OR (High Blood Pressures[Title/Abstract]))

 #4 "Weight Loss"[Majr]

 #5 ((((((Loss, Weight[Title/Abstract]) OR (Losses, Weight[Title/Abstract])) OR (Weight Losses[Title/Abstract])) OR (Weight Reduction[Title/Abstract])) OR (Reduction, Weight[Title/Abstract])) OR (Reductions, Weight[Title/Abstract])) OR (Weight Reductions[Title/Abstract]) 

 #6 ("Weight Loss"[Majr]) OR (((((((Loss, Weight[Title/Abstract]) OR (Losses, Weight[Title/Abstract])) OR (Weight Losses[Title/Abstract])) OR (Weight Reduction[Title/Abstract])) OR (Reduction, Weight[Title/Abstract])) OR (Reductions, Weight[Title/Abstract])) OR (Weight Reductions[Title/Abstract]))

 #7 randomized controlled trial[Publication Type] OR randomized[Title/Abstract] OR placebo[Title/Abstract]

 #8 ((("Hypertension"[Mesh]) OR ((((Blood Pressure, High[Title/Abstract]) OR (Blood Pressures, High[Title/Abstract])) OR (High Blood Pressure[Title/Abstract])) OR (High Blood Pressures[Title/Abstract]))) AND (("Weight Loss"[Majr]) OR (((((((Loss, Weight[Title/Abstract]) OR (Losses, Weight[Title/Abstract])) OR (Weight Losses[Title/Abstract])) OR (Weight Reduction[Title/Abstract])) OR (Reduction, Weight[Title/Abstract])) OR (Reductions, Weight[Title/Abstract])) OR (Weight Reductions[Title/Abstract])))) AND (randomized controlled trial[Publication Type] OR randomized[Title/Abstract] OR placebo[Title/Abstract])

Embase, and Cochrane Library databases #1 ("Weight Loss"[Majr]) OR (((((((Loss, Weight[Title/Abstract]) OR (Losses, Weight[Title/Abstract])) OR (Weight Losses[Title/Abstract])) OR (Weight Reduction[Title/Abstract])) OR (Reduction, Weight[Title/Abstract])) OR (Reductions, Weight[Title/Abstract])) OR (Weight Reductions[Title/Abstract]))

Commment 3: Table 2 is incomprehensible, I suggest redoing it.Meta-analyses need to be redone: plot by outcome and create subgroups for the interventions included in the analysis.

Authors’ Response: We think this is an excellent suggestion. The tables and Meta-analysis results have then been redrawn.

Basic information of the included literature Table

Author,Year Cases Age (years) Treatment Intervention period /month Outcome indicator

 T C T C T C 

Mori TA,2000 [15]

26 14 50.8±2.0 50.6±1.4 EPA/DHA Placebo 1.5 SBP,DBP,Bodyweight,BMI,TNF-α,LDL, HDL,TC,TG,Glucose,CRP,TAS

Pierce GL,2008 [16]

26 14 49.2±2.5 40.8±3.3 Weight Loss Untreated 4 SBP,DBP,Heart rate, Body weight,BMI,ET-1,TNF-α,LDL,HDL,TC,TG,Glucose

Farpour-Lambert NJ, 2009[17]

22 22 9.1±1.4 8.8±1.6 Obese Exercise Untreated 3 SBP,DBP, Body weight,BMI, LDL,HDL,TC,TG,FBF

Bogdanski P,2012 [13]

28 28 49.2±8.8 51.5±7.4 Green tea extract Placebo 3 SBP,DBP, Body weight,BMI, LDL,HDL,TC,TG, Glucose ,FBF

Croymans DM,2014 [18]

28 8 22.0 21.5 resistance training Untreated 3 SBP,DBP,Heart rate,Body weight,BMI,sICAM-1,sVCAM-1,LDL,HDL,TG,CRP

Brüll V,2015 [14]

68 68 7.4±10.5 59.6±4.3 Quercetin Placebo 1.5 SBP,DBP,Heart rate,Body weight,BMI,sICAM-1,sVCAM-1, LDL,HDL,TG,CRP

Kianbakht S,2019[20]

50 50 57±6 61±8 V. arctostaphylos berry extract Placebo 3 SBP,DBP, Heart rate, sICAM-1,sVCAM-1,ET-1,LDL,HDL,TG,CRP

Wong A,2020[19]

14 14 22±1 23±1 Placebo Untreated 3 SBP,DBP,BMI

Note：T: The treatment group； C: Control group；

Remapping has been carried out depending on the intervention, there are two types of lifestyle and dietary supplements.

Fig3. Forest plot of primary outcome indicators(I)

(A) SBP Forest plot of primary outcome indicators (B) DBP Forest plot of primary outcome indicators (C) Heart rate Forest plot of primary outcome indicators 

Fig 4.Forest plot of primary outcome indicators(II)

(A) Boby weight Forest plot of primary outcome indicators (B) BMI Forest plot of primary outcome indicators (C) Glucose Forest plot of primary outcome indicators

Fig 5.Forest plot of primary outcome indicators(III)

(A) TNF-α Forest plot of primary outcome indicators (B) Endothelin-1 Forest plot of primary outcome indicators (C) sICAM-1Forest plot of primary outcome indicators(D) sVCAM-1 Forest plot of primary outcome indicators

Fig 6.Forest map secondary outcome indicators

(A) TG Forest map secondary outcome indicators (B) TC Forest map secondary outcome indicators (C) HDL Forest map secondary outcome indicators (D) LDL Forest map secondary outcome indicators

Fig 7.Forest map secondary outcome indicators

(A) CRP Forest map secondary outcome indicators (B) hs-CRP Forest map secondary outcome indicators (C) TAS Forest map secondary outcome indicators

Commment 4: GRADE Table 3 is incomprehensible and incomplete: it is best to redo it using GRADE Pro.

Authors’ Response: Your suggestion really means a lot to us，already redrawn with GRADE Pro.

Fig 10.GRADE levels of Evidence

Commment 5: The authors mixed the effect of totally different interventions (life-style and dietary supplements) trying to drive a common conclusion. This makes not sense from a clinical point of view and the conclusion are supported by statistical analysis, but are not usefult.

Authors’ Response: First of all, thank you very much for your valuable comments, which point out the shortcomings of this paper. We have now compared the observed indicators by lifestyle and dietary supplements in a subgroup analysis, and it is clear from the indicators analysed that the effect of lifestyle changes in lowering blood pressure and improving the endothelium is more pronounced. A European Society of Hypertension position document has clearly stated the status of Nutraceuticals and blood pressure control, the aim of this paper is to make more people aware of the benefits of non-pharmacological treatments and to promote them so that more people can benefit.

Commment 6: Figure 1: The authors should explain what do they mean by "study design is not rigorous". The authors should also assess for the presence of publication bias in the analysis by performing the Egger and Begg's tests.

Authors’ Response: Firstly, there are limitations in the six papers, all of which relate to the endothelium of non-pharmacological obese hypertensive patients; The first article: no randomised sequential analysis, no blinding and too small a sample size; The second article: no blinded evaluation of the results and relatively limited participation in the study; The third article: no hidden allocation of participants and no comparison of the treatment of the trial and control groups. In the fourth article, dietary therapy was used for both the test and control groups, and no comparison was made between the normal group; In the fifth and sixth articles, no control observations were made, especially in the sixth article, which used different groups of thin and fat people, with a large margin of error, and no post-treatment observation was made on the same person. The following six articles are DOI:DOI: 10.1016/s0895-7061(01)02322-6、DOI: 10.1080/07315724.2016.1194236、DOI: 10.1038/jhh.2009.58 、DOI: 10.3109/08037051.2014.976979、

DOI: 10.1016/j.numecd.2010.03.003 、DOI: 10.1016/s0895-7061(01)02191-4

The Egger and Begg's tests were not biased because the third, fifth, and sixth articles were not subjected to controlled observations.

The following are the first, second and fourth Egger and Begg's tests 

由于Begg's tests P=1; Egger tests P=0.29, not statistically significant when p>0.05.

Commment 7: The limitations of the study should be further and more deeply discussed.

Authors’ Response: Only life-styl and dietary supplements were analyzed for non-pharmacological treatment, and there was a lack of observation and analysis for vegetables, low-fat dairy products, and non-nutritional supplements.There are relatively few studies on TNF-α, Endothelin-1 and other endothelium-related indicators of hypertension, and the effects are not good enough to further explore the extent to which their non-pharmacological effects on the vascular endothelium of obese hypertensive patients.

Commment 8: The authors should highly consider to include in their manuscript the references doi: 10.1007/s40292-021-00474-6, doi: 10.33963/KP.15468, doi: 10.1097/HJH.0000000000002353 and doi: 10.1007/s40292-018-0296-6

Authors’ Response: These papers are of great value to this paper and have been added to this paper, Lines 54-56 and 264-267, respectively, with references 8, 9 and 21, 22.

Commment 9: Please ensure that your manuscript meets PLOS ONE's style requirements, including those for file naming. 

Authors’ Response: Already modified according to the PLOS ONE's style requirements.

Commment 10: Thank you for stating the following financial disclosure: "NO".At this time, please address the following queries:a) Please clarify the sources of funding (financial or material support) for your study. List the grants or organizations that supported your study, including funding received from your institution. b) State what role the funders took in the study. If the funders had no role in your study, please state: “The funders had no role in study design, data collection and analysis, decision to publish, or preparation of the manuscript.”c) If any authors received a salary from any of your funders, please state which authors and which funders.d) If you did not receive any funding for this study, please state: “The authors received no specific funding for this work.Please include your amended statements within your cover letter; we will change the online submission form on your behalf.

Authors’ Response:Funding:Jinan Science and Technology Bureau Clinical Medical Science and Technology Innovation Program (No. 202134024), YC was involved in the conception and design of the study and provided scientific oversight.

Commment 11:Thank you for stating the following in your Competing Interests section: "NO authors have competing interestsEnter: The authors have declared that no competing interests exist."Please complete your Competing Interests on the online submission form to state any Competing Interests. If you have no competing interests, please state "The authors have declared that no competing interests exist.", as detailed online in our guide for authors at http://journals.plos.org/plosone/s/submit-now.This information should be included in your cover letter; we will change the online submission form on your behalf.

Authors’ Response: "The authors have declared that no competing interests exist."

Commment 12:Please upload a copy of Figure 9, to which you refer in your text on page 13. If the figure is no longer to be included as part of the submission please remove all reference to it within the text.

Authors’ Response:Fig1.:SBP Fig2.:DBP Fig3.:TNF-αFig4.:Endothelin-1 Fig5.: sICAM-1 Fig6.:sVCAM-1

Fig1.

Fig2.

Fig3. Fig4.

Fig5.

Fig6.

Attachment Submitted filename: Response to Reviewers.docx Click here for additional data file.

# Peer review: Decision Letter 1

**DOI:** 10.1371/journal.pone.0279582.r003

**Authors:** Marcus Tolentino Silva

12 Dec 2022

Metabolic status and vascular endothelial structure in obese hypertensive patients treated with non-pharmacological therapies: a systematic review and meta-analysis

PONE-D-22-16167R1

Dear Dr. Cheng,

We’re pleased to inform you that your manuscript has been judged scientifically suitable for publication and will be formally accepted for publication once it meets all outstanding technical requirements.

Within one week, you’ll receive an e-mail detailing the required amendments. When these have been addressed, you’ll receive a formal acceptance letter and your manuscript will be scheduled for publication.

An invoice for payment will follow shortly after the formal acceptance. To ensure an efficient process, please log into Editorial Manager at http://www.editorialmanager.com/pone/, click the 'Update My Information' link at the top of the page, and double check that your user information is up-to-date. If you have any billing related questions, please contact our Author Billing department directly at authorbilling@plos.org.

If your institution or institutions have a press office, please notify them about your upcoming paper to help maximize its impact. If they’ll be preparing press materials, please inform our press team as soon as possible -- no later than 48 hours after receiving the formal acceptance. Your manuscript will remain under strict press embargo until 2 pm Eastern Time on the date of publication. For more information, please contact onepress@plos.org.

Kind regards,

Marcus Tolentino Silva

Academic Editor

PLOS ONE

Additional Editor Comments (optional):

Reviewers' comments:

Reviewer's Responses to Questions

**Comments to the Author**

1. If the authors have adequately addressed your comments raised in a previous round of review and you feel that this manuscript is now acceptable for publication, you may indicate that here to bypass the “Comments to the Author” section, enter your conflict of interest statement in the “Confidential to Editor” section, and submit your "Accept" recommendation.

Reviewer #1: All comments have been addressed

Reviewer #2: All comments have been addressed

**********

2. Is the manuscript technically sound, and do the data support the conclusions?

The manuscript must describe a technically sound piece of scientific research with data that supports the conclusions. Experiments must have been conducted rigorously, with appropriate controls, replication, and sample sizes. The conclusions must be drawn appropriately based on the data presented. 

Reviewer #1: (No Response)

Reviewer #2: Yes

**********

3. Has the statistical analysis been performed appropriately and rigorously? 

Reviewer #1: (No Response)

Reviewer #2: Yes

**********

4. Have the authors made all data underlying the findings in their manuscript fully available?

The PLOS Data policy requires authors to make all data underlying the findings described in their manuscript fully available without restriction, with rare exception (please refer to the Data Availability Statement in the manuscript PDF file). The data should be provided as part of the manuscript or its supporting information, or deposited to a public repository. For example, in addition to summary statistics, the data points behind means, medians and variance measures should be available. If there are restrictions on publicly sharing data—e.g. participant privacy or use of data from a third party—those must be specified.

Reviewer #1: (No Response)

Reviewer #2: Yes

**********

5. Is the manuscript presented in an intelligible fashion and written in standard English?

PLOS ONE does not copyedit accepted manuscripts, so the language in submitted articles must be clear, correct, and unambiguous. Any typographical or grammatical errors should be corrected at revision, so please note any specific errors here.

Reviewer #1: (No Response)

Reviewer #2: Yes

**********

6. Review Comments to the Author

Please use the space provided to explain your answers to the questions above. You may also include additional comments for the author, including concerns about dual publication, research ethics, or publication ethics. (Please upload your review as an attachment if it exceeds 20,000 characters)

Reviewer #1: The authors have considered the reviewers’ suggestion and improved the paper accordingly. I’ve no further comments on it.

Reviewer #2: Dear Editor,

I carefully read the revised version of the manuscript that is significantly improved in comparison with the previous one. I warmly recommend its publication in the Journal.

**********

7. PLOS authors have the option to publish the peer review history of their article (what does this mean?). If published, this will include your full peer review and any attached files.

If you choose “no”, your identity will remain anonymous but your review may still be made public.

**Do you want your identity to be public for this peer review?** For information about this choice, including consent withdrawal, please see our Privacy Policy.

Reviewer #1: No

Reviewer #2: No

**********

# Editor report: Acceptance letter

**DOI:** 10.1371/journal.pone.0279582.r004

**Authors:** Marcus Tolentino Silva

16 Dec 2022

PONE-D-22-16167R1 

Metabolic status and vascular endothelial structure in obese hypertensive patients treated with non-pharmacological therapies: a systematic review and meta-analysis 

Dear Dr. Cheng:

I'm pleased to inform you that your manuscript has been deemed suitable for publication in PLOS ONE. Congratulations! Your manuscript is now with our production department. 

If your institution or institutions have a press office, please let them know about your upcoming paper now to help maximize its impact. If they'll be preparing press materials, please inform our press team within the next 48 hours. Your manuscript will remain under strict press embargo until 2 pm Eastern Time on the date of publication. For more information please contact onepress@plos.org.

If we can help with anything else, please email us at plosone@plos.org. 

Thank you for submitting your work to PLOS ONE and supporting open access. 

Kind regards, 

PLOS ONE Editorial Office Staff

on behalf of

Dr. Marcus Tolentino Silva 

Academic Editor

PLOS ONE
